# The risks of *RELN* polymorphisms and its expression in the development of otosclerosis

**DOI:** 10.1371/journal.pone.0269558

**Published:** 2022-06-03

**Authors:** Saurabh Priyadarshi, Kirtal Hansdah, Neha Singh, Amal Bouzid, Chinmay Sundar Ray, Khirod Chandra Panda, Narayan Chandra Biswal, Ashim Desai, Jyotish Chandra Choudhury, Adel Tekari, Saber Masmoudi, Puppala Venkat Ramchander

**Affiliations:** 1 Institute of Life Sciences, Nalco Square, Chandrasekharpur, Bhubaneswar, India; 2 Laboratory of Molecular and Cellular Screening Processes, Centre of Biotechnology of Sfax, University of Sfax, Sfax, Tunisia; 3 Department of Ear, Nose, and Throat (ENT), Shrirama Chandra Bhanj (SCB) Medical College & Hospital, Cuttack, India; 4 Ear, Nose, and Throat (ENT) Unit, Capital Hospital, Unit VI, Bhubaneswar, India; 5 Dr. ABR Desai Ear, Nose and Throat (ENT) Clinic and Research Centre, Mumbai, India; 6 Department of Forensic Medicine & Toxicology (FMT), Shrirama Chandra Bhanj (SCB) Medical College & Hospital, Cuttack, India; UCSI University, MALAYSIA

## Abstract

Otosclerosis (OTSC) is the primary form of conductive hearing loss characterized by abnormal bone remodelling within the otic capsule of the human middle ear. A genetic association of the *RELN* SNP rs3914132 with OTSC has been identified in European population. Previously, we showed a trend towards association of this polymorphism with OTSC and identified a rare variant rs74503667 in a familial case. Here, we genotyped these variants in an Indian cohort composed of 254 OTSC cases and 262 controls. We detected a significant association of rs3914132 with OTSC (OR = 0.569, 95%CI = 0.386–0.838, p = 0.0041). To confirm this finding, we completed a meta-analysis which revealed a significant association of the rs3914132 polymorphism with OTSC (Z = 6.707, p<0.0001) across different ethnic populations. Linkage analysis found the evidence of linkage at *RELN* locus (LOD score 2.1059) in the OTSC family which has shown the transmission of rare variant rs74503667 in the affected individuals. To understand the role of RELN and its receptors in the development of OTSC, we went further to perform a functional analysis of RELN/reelin. Here we detected a reduced *RELN* (p = 0.0068) and *VLDLR* (p = 0.0348) mRNA levels in the otosclerotic stapes tissues. Furthermore, a reduced reelin protein expression by immunohistochemistry was confirmed in the otosclerotic tissues. Electrophoretic mobility shift assays for rs3914132 and rs74503667 variants revealed an altered binding of transcription factors in the mutated sequences which indicates the regulatory role of these variations in the *RELN* gene regulation. Subsequently, we showed by scanning electron microscopy a change in stapes bone morphology of otosclerotic patients. In conclusion, this study evidenced that the rare variation rs74503667 and the common polymorphism rs3914132 in the *RELN* gene and its reduced expressions that were associated with OTSC.

## Introduction

Hearing loss in humans represents a real health burden as it reduces the quality of life and leads to subsequent social isolation. Otosclerosis (OTSC) is the main form of conductive hearing impairment, characterized by an ossification of the stapes footplates which hinders its mobility leading to progressive hearing loss in humans. The typical age of onset for OTSC is in the third decade and the disease complexity progresses with age. Clinical OTSC has a prevalence of 0.3–0.4% in European and 0.4–0.8% in the non-European populations [1]. In India, epidemiological studies suggest that OTSC is a common cause of acquired hearing loss, affecting with higher incidence in the population [2]. OTSC has been widely accepted as a complex and multifactorial disorder, where both genetic and environmental factors are involved in the etiology. Family and twin studies have indicated that genetic factor plays a significant role in the disease manifestation [3]. Despite intensive study on OTSC, till date ten monogenic loci has been mapped but have not yet identified the causal genes [4,5]. Several case-control studies reported a significant association of SNPs in *COL1A1 [6,7]*, *TGF-β1 [8,9]*, *BMP2 [10], BMP4 [7,10]* and *OPG [11,12]* genes with OTSC in different populations. Recently, high throughput sequencing has spotted certain pathogenic variants in *MEPE*, *ACAN* and *SERPINF1* genes with unsettled pathogenicity [5,13].

A genome wide association study in European population has identified two regions on chr7q22.1 and chr11q13.1 to be associated with OTSC. The chr7q22.1 region is located in the *RELN* gene harbouring an intronic SNP rs3914132 was found to be strongly associated with OTSC [14]. Multiple studies have replicated this association in different populations [1,15]. Some of the studies were underpowered for detection of the association with this SNP [16,17].

The RELN (OMIM:600514) gene, encoding reelin protein is considered an important decoy receptor that regulate essentially primary neuronal and neuroglial interactions. Reelin is expressed exclusively by neural tissues [18,19] and is therefore reported in the pathogenesis of several brain disorders [20–22]. Reelin has a serine-protease activity that is important for the modulation of cell adhesion [23]. It binds to very low density lipoprotein receptor (VLDLR) and low density lipoprotein receptor-related protein 8 (LRP8) resulting in phosphorylation of intracellular protein Dab1 which is essential for effective reelin signalling cascade. Genetic changes in *RELN* gene can result in abnormal reelin signalling leading to different pathological conditions such as bipolar disease, schizophrenia and autism [24–26].The physiological significance of the reelin in bone metabolism is generally not well understood. A recent study by Dou et al. [27] evidenced that reelin is a potent regulator of bone formation and that reelin depletion regulated osteolysis and osteogenesis balance. Osteocytes, the mechanosensing cells of the bone, express high levels of reelin [28–30]. In addition, a site-specific expression of the reelin in limb and skull in adult animal bone cells and during embryonic development [31]. *RELN* expression has been found in the mouse inner ear structures [14] and an evidence of distinct reelin expression in human otosclerotic stapes tissues [32] supporting its involvement in OTSC pathogenesis.

To date, although accumulating data have documented the association of *RELN* SNP rs3914132 and OTSC risk, its role in the progression of OTSC remains inconclusive. Hence, in the present study, we investigated the genetic association of *RELN* variants with OTSC in adequate sample size, its expression levels and its role in stapes tissues of diseased condition.

## Materials and methods

### Study participants

The case group consisted of 254 (169 men and 85 women) unrelated non-syndromic OTSC patients (mean age ± SD of 40.50 ± 13.62 years) and a multigenerational OTSC family consecutively enrolled from Ear, Nose and Throat (ENT) units of Capital hospital, Bhubaneswar and SCB medical college, Cuttack, Odisha, India. These patients were diagnosed based on family history, otoscopy, pure tone audiometry, and impedance testing. Pure tone audiometry was performed in a double walled soundproof room using standard procedures. The frequencies tested for air conduction were 125, 250, 500, 1000, 2000, 4000, and 8000 Hz and for bone conduction were 250, 500, 1000, 2000, and 4000 Hz. The controls contained 262 (183 men and 79 women) ethnic and sex-matched healthy individuals (mean age ± SD of 33 ± 10.76 years). The control group individuals were randomly selected without any history of hearing disorder, in particular otosclerosis, or any other metabolic bone-related diseases. The extended multigenerational OTSC family includes 12 informative individuals: 6 affected, 5 unaffected and 1 with uncertain disease status. The clinical description of the family members are listed in S1 Table.

Peripheral venous blood (5ml) was collected from all the individuals in EDTA vacutainers and store at 4°C. The stapes tissues (N = 52) were collected from the patients who undergone stapedectomy. The controls stapes (N = 39) were obtained from the cadavers immediate post-mortem. Three incus bones from patients undergoing surgery for middle ear cholesteatoma were also applied as additional controls. All the tissues collected were placed into RNAlater (Qiagen, GmbH, Hilden, Germany) to stabilize and protect RNA in intact until further use.

This study was approved by the Institutional Ethical Committees of Institute of Life Sciences, Bhubaneswar and SCB Medical College, Cuttack and the methods were carried out in accordance with approved guidelines. Written informed consent was obtained from all participants of this study.

### Direct DNA sequencing of *RELN* intron 2 region

DNA was extracted from venous blood samples using rapid non-enzymatic method [33]. PCR amplification was performed using the primers and conditions as previously described [16]. All the amplified products were purified and sequenced using BigDye Terminator v3.1 Cycle Sequencing Kit; (Applied Biosystems, Inc., Foster City, CA, USA) in forward and reverse directions with the same primers used for amplification.

### Meta-analysis of rs3914132 SNP

Meta-analysis was performed to calculate the common genetic effect size of the SNP rs3914132 in *RELN* gene associated with OTSC across different populations following Comprehensive Meta-Analysis (CMV) software (version 1.2; Biostat Inc, Englewood, USA) guideline [34]. The criteria followed includes- a) sample size of cases and controls, and b) p-value obtained from the studies. The heterogeneity between studies (I^2^) was evaluated by fixed-effects model (I^2^< 50%) or a random-effects model (I^2^>50%) [35,36]. A p-value <0.05 was considered as statistically significant in any of the effect model. To assess the potential publication bias, Egger regression test was used and sensitivity analysis was estimated by one study removal in each genetic model [37–39].

### Microsatellites genotyping

Genotyping was performed to the known OTSC loci using STR markers for each of the reported locus. Additionally, three markers (D7S2509, D7S2504 and D7S796) were selected at 7q22.1 for mapping the *RELN* locus. Fluorescently labelled primers (FAM or HEX) were used for genotyping (S2 Table). PCR was performed using Type-it microsatellite PCR kit (Qiagen, GmbH, Hilden, Germany). For allele size determination multiplexing of amplified products was performed in pooling plate in a manner that avoided mixing of PCR products of the same size labelled with same dye. The PCR products (0.2 μl) of three different markers labelled with one of the fluorescent dye FAM or HEX together with 9.2μl of Hi-Di formamide (Applied Biosystems, Inc., Foster City, CA, USA) and 0.2μl of internal size standard ROX or LIZ (Applied Biosystems, Inc., Foster City, CA, USA) were aliquoted in a 96 well plate. The samples were denatured at 95°C for 10 min and immediately snap cooled for 10 min at -20°C. The denatured samples consisting of dye labeled PCR products and size standard fragments were loaded on eight capillary 3500 genetic analyzer and separated based on size and charge as they move through the POP-7^TM^ polymer filled in capillaries. Fragment analysis was carried out on ABI 3500 Genetic Analyzer (Applied Biosystems, Inc., Foster City, CA, USA). Allele sizes were determined using GeneMapper software 1.3(Applied Biosystems, Inc., Foster City, CA, USA).

### Linkage analysis and haplotype construction

Parametric and nonparametric two-point and multi-point linkage analyses were performed using the programs SuperLink v1.4 and GeneHunter of easyLINKAGE Plus v4.01 software package [40]. Linkage analysis was carried out assuming different modes of inheritance, dominant versus recessive, different penetrance levels (40–90%) and 1% phenocopy rate. The final linkage parameter for OTSC family was chosen assuming that the disease is inherited in an autosomal dominant pattern with a gene frequency of 0.01% and a phenocopy rate of 1%. Haplotypes were constructed using HaploPainter software [41].

### Expression analysis of *RELN/VLDLR/LRP8* genes

Extraction of total RNA from tissues and cDNA synthesis was carried out following previously described methods [42]. RT-PCR was performed for the *RELN*, *VLDLR* and *LRP8* with gene specific primers (S3 Table) using Go green PCR master mix (Promega, Madison, USA). The expressions were quantified by using QuantiTect SYBER Green RT-PCR Kit (Qiagen, GmbH, Hilden, Germany) on ABI StepOne Real-Time PCR system (Applied Biosystems, Inc., Foster City, CA, USA). Each sample was assayed twice in triplicate. The comparative ΔΔC_T_ method was used to quantify the mRNA relative level to the average expression of the *18S rRNA*, an endogenous control for data normalization. The resulted data of two independent analyses for each parameter were averaged and the relative expression levels were presented as the relative fold change analysed using unpaired two-tailed Student’s t test.

### Electrophoretic mobility shift assay

Further, we assessed the functional consequence of the 1-bp substitution by electrophoretic mobility shift assay (EMSA) to detect the altered binding of transcription factors (TFs) in the mutated sequence. EMSA was conducted following the Hellman and Fried protocol [43]. All reactions included double stranded, ^32^P-labeled, oligonucleotides probes corresponding to wild type and mutated *RELN* gene variants rs3914132 and rs74503667. Nuclear protein extract from SH-SY5Y cells were incubated with ^32^P-labeled probe in binding buffer at room temperature before loading on to 8% polyacrylamide gel. The samples were then electrophoresed at 160V for 4 hours in cold condition. After migration, the gel was dried and exposed overnight at -20°C. Visualization was carried out using a Kodak infrared Imager system.

### Scanning electron microscopy

Scanning electron microscopy (SEM) was performed to find the basic architectural differences between the otosclerotic and normal stapes. The stapes was fixed, decalcified, embedded, and sectioned in to 10μm slides at -25°C by cryomicrotome (Leica, CM1850-1-1, Germany). Sections were stored in 0.1M PBS containing 0.03% sodium azide at 4°C. Samples were dehydrated in a series of ethanol washes (10%, 25%, 50%, 70%, 90%, and 100%), critical point dried, and sputter coated with gold palladium before imaging with a ZEISS EVO 18 (Carl Zeiss SMT Ltd, Cambridge, UK). All steps were carried out as previously described [44].

### Immunofluorescence

The reelin protein expression was determined by immunofluorescence assay following previous report [45]. The case and control stapes bones were decalcified followed by dehydration in 80 to 100% ethanol, cleared in xylene and embedded in paraffin. Stapes sections (5μm) were deparaffinized in xylene and rehydrated in graded ethanol. The sections were boiled in antigen unmasking reagent (Vectastain ABC Kit, Vector Laboratories) and blocked with horse serum for 30 mins. Then, the sections were incubated overnight with reelin primary antibody (1:100, #sc32554; Santa Cruz) at 4°C in a humidified chamber. After washing, sections were incubated with secondary antibody conjugated with Alexa fluor 594 (1:500, A11080; Invitrogen) at room temperature for 45 mins in dark. The slides were mounted with SlowFade Gold Antifade reagent with DAPI (Life Technologies Corporation, USA). Next, the slides were visualized, and images were captured under a confocal microscope (Leica TCS SP8 STED).

### Statistical analysis

To identify the association of *RELN* variants with OTSC, genotype and allele frequency were calculated by the direct counting method. Power calculations were conducted with the genetic power calculator to estimate the sample size for this study [46]. The deviation from Hardy Weinberg equilibrium (HWE) was determined using a goodness-of-fit Chi-squared test to compare the observed genotype frequencies with the expected frequencies, from the control groups using HWE calculator [47]. The association testing between genotypes and phenotype was performed by Cochran-Armitage trend test using SNPalyze V8.0.2. software (Dynacom, Chiba, Japan). The Fisher’s exact test was used to assess the differences in allele frequencies between the case and control groups. Odds ratio (OR) at 95% confidence interval (CI) was calculated to check the association of genotypes and alleles with OTSC. The statistical analysis was performed using GraphPad prism (version 8.0 from Windows, San Diego, CA, USA). Bonferroni correction for multiple testing of four SNPs (0.05/4 = 0.0125) was used and p<0.0125 was considered statistically significant. All other statistical analysis were described in the corresponding sections.

## Results

### *RELN* gene SNP rs3914132 is associated with OTSC

**Fig 1:**
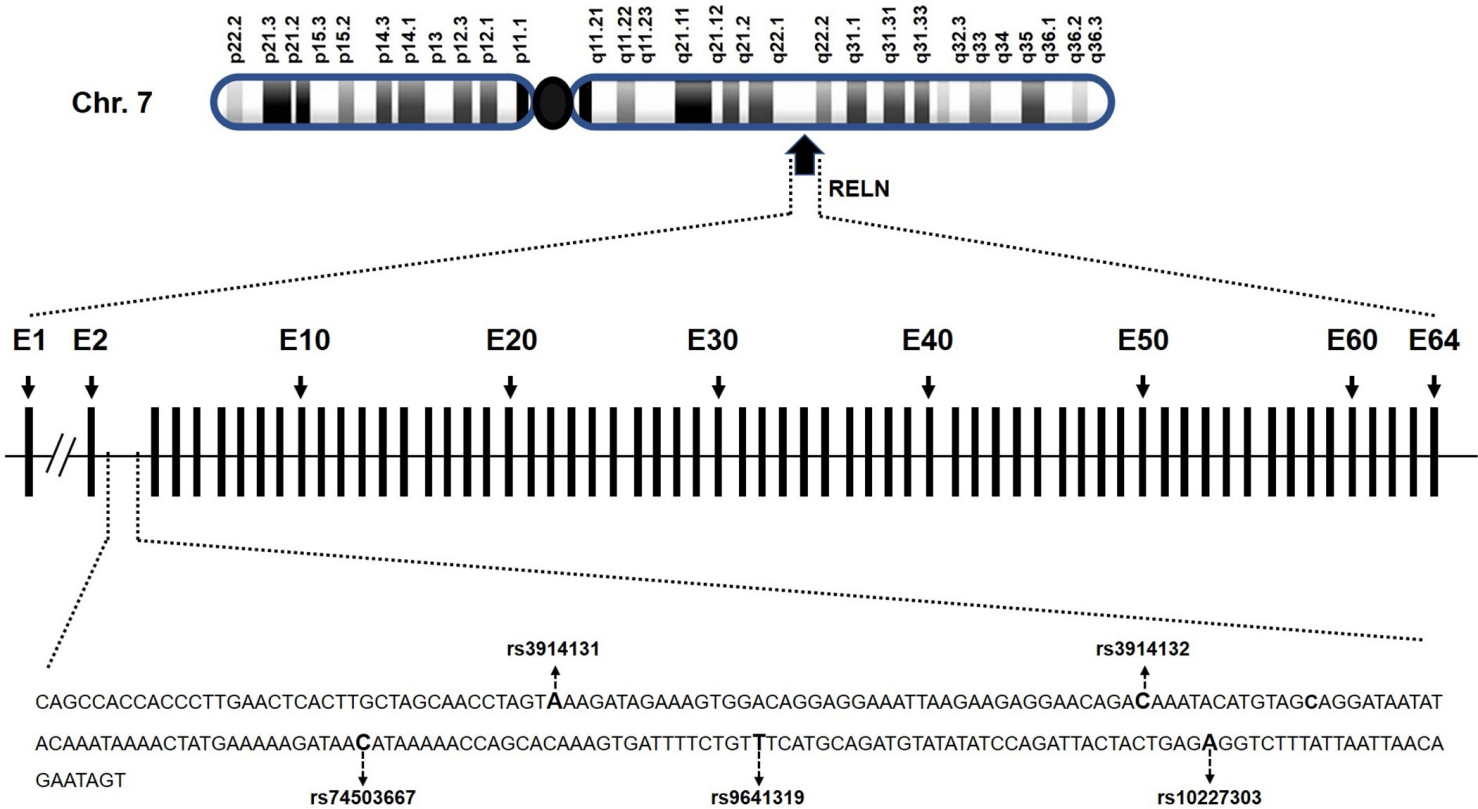
Genomic location and organization of *RELN* gene. *RELN* gene consists of 64 exons (E1-E64). Four known polymorphisms were detected in intron 2 region of otosclerosis cases and controls. A rare variant rs74503667 was found in a familial case of otosclerosis.

To explore the genetic association of the *RELN* SNP rs3914132 with OTSC, we sequenced intron 2 of *RELN* gene in 254 cases and 262 controls. Sequence analysis revealed four known SNPs rs3914131, rs3914132, rs9641319, rs10227303 and a rare variant rs74503667 (Fig 1).

The genotype frequencies of all the common SNPs were in Hardy-Weinberg expectation in both the groups. The calculated allele and genotype frequencies of *RELN* variants are shown in Table 1. A highly significant difference was observed for rs3914132 in the allele count of the cases 46/508 (0.09) and the controls 78/524 (0.15). Fisher’s exact test confirmed the association between this SNP and OTSC (p = 0.0041, OR = 0.569, 95%CI = 0.386–0.838). Association testing with genotype frequency of this variation showed a consistent association with OTSC (p = 0.0045, OR = 0.569, 95%CI = 0.387–0.338). The association remained significant (p<0.0125) even after Bonferroni correction. The decreased minor allele frequency (MAF) ‘C’ in cases (0.09) compared to controls (0.15) indicates its protective role in OTSC development. We also found co-segregation (complete linkage disequilibrium) of rs3914131 with rs3914132 showing similar genotype and allele frequencies.

**Table 1:** Allele and Genotype frequencies distribution of *RELN* gene polymorphisms in non-syndromic otosclerosis cases and controls.

SNP	Genotype Allele	Cases (N = 254)	Controls (N = 262)	OR (95%CI)	p value
rs3914131	GG	211 (83.07)	190 (72.51)	0.569 (0.387–0.338)	**0.0045^a^**
	AG	40 (15.74)	66 (25.19)		
	AA	03 (1.18)	06 (2.29)		
	G	462 (0.91)	446 (0.85)	0.569 (0.386–0.838)	**0.0041^b^**
	A	46 (0.09)	78 (0.15)		
rs3914132	TT	211 (83.07)	190 (73.51)	0.569 (0.387–0.338)	**0.0045^a^**
	CT	40 (15.74)	66 (25.19)		
	CC	03 (1.18)	06 (2.29)		
	T	462 (0.91)	446 (0.85)	0.569 (0.386–0.838)	**0.0041^b^**
	C	46 (0.09)	78 (0.15)		
rs9641319	TT	97 (38.18)	104 (39.69)	1.051 (0.817–1.352)	0.6990^a^
	TC	120 (47.24)	122 (46.56)		
	CC	37 (14.56)	36 (13.74)		
	T	314 (0.62)	330 (0.63)	1.051 (0.816–1.352)	0.7004^b^
	C	194 (0.38)	194 (0.37)		
rs10227303	AA	230 (90.55)	235 (89.69)	1.074 (0.624–1.849)	0.8060^a^
	AT	20 (7.87)	27 (10.31)		
	TT	04 (1.57)	00 (0.00)		
	A	480 (0.94)	497 (0.95)	1.074 (0.623–1.849)	0.8899^b^
	T	28 (0.06)	27 (0.05)		

p< 0.0125 was considered as significant. Significant p values are marked in bold. OR = odds ratio, CI = confidence interval. The SNPs remained the evidence of significant association even after Bonferroni correction for multiple testing (0.05/4 = 0.0125).

^**a**^The association testing between genotypes and phenotype was performed by the Cochran-Armitage trend test using SNPalyze V8.0.2.

^**b**^Fisher’s exact test was used to assess the differences in allele frequencies between the case and control groups.

**Fig 2:**
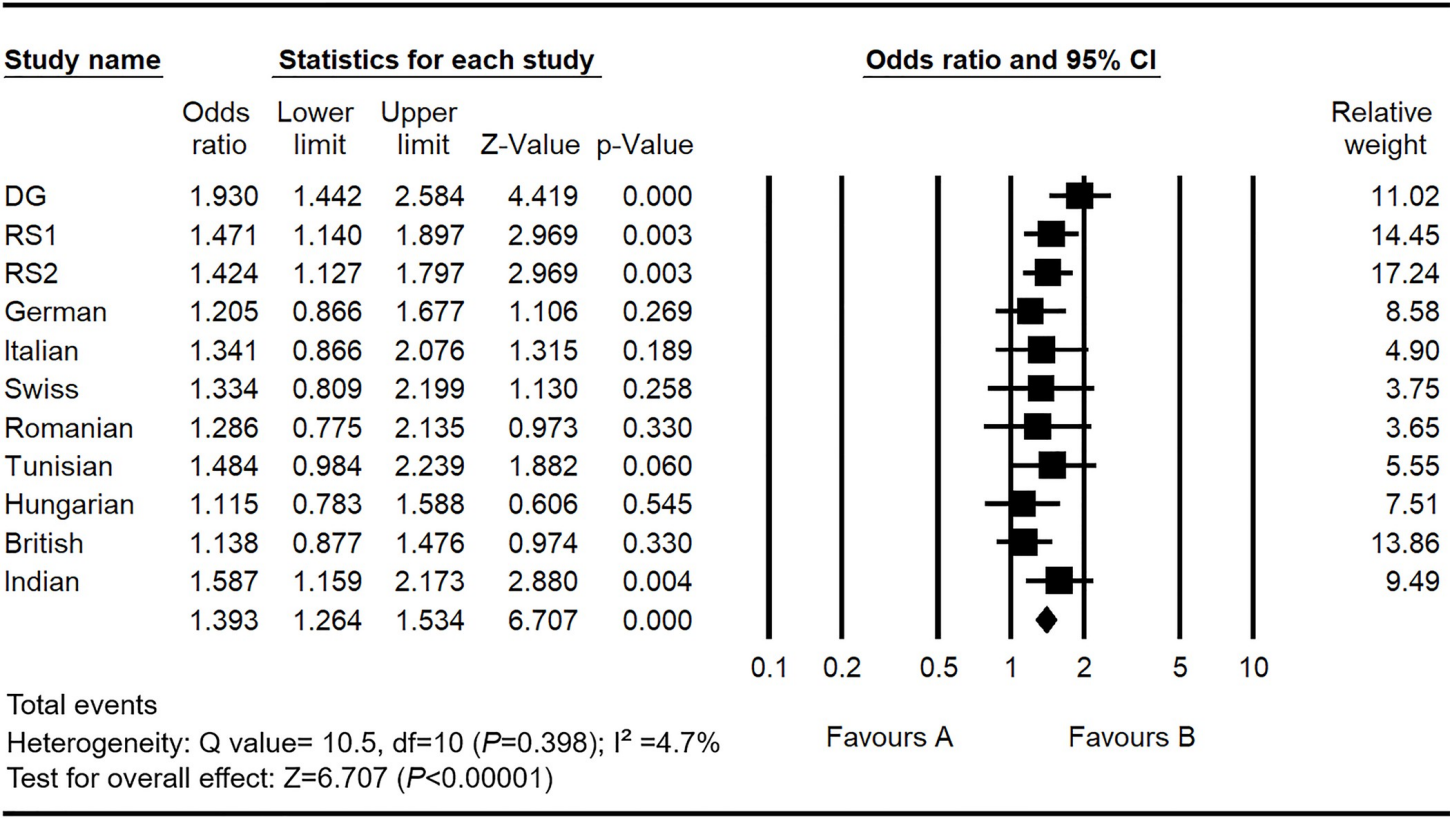
Meta-analysis plot for rs3914132 SNP in *RELN* gene. The point estimates of the odds ratio (OR) for each study are listed. Graphically, the 95% confidence interval for each study is given by a horizontal line, and the point estimate is given by a square whose height is inversely proportional to the standard error of the estimate. The common OR, is drawn as a diamond with horizontal limits at the confidence limits and width is inversely proportional to its standard error. Populations included in this meta-analysis are provided from the genome wide association study (DG: Discovery group, RS1: Replication Set 1, RS2: Replication Set 2) (Schrauwen et al., 2009), a follow up study in 4 European populations (GER: German, ITA: Italian, CH: Swiss, ROM: Romanian) (Schrauwen et al., 2010), Tunisian, Hungarian & British populations (Khalfallah et al., 2010; Sommen et al., 2014 and Mowat et al., 2018) and Indian population from the present study.

Multiple population studies of the rs3914132 SNP with OTSC allowed us to conduct a meta-analysis using a reasonable sample size (Fig 2). The cumulative study population composed of 2670 cases and 2812 controls was analysed (S4 Table). No significant heterogeneity was found between these studies (I^2^ = 4.7%; p = 0.398), hence, a fixed effect procedure was performed to provide a pooled OR. The meta-analysis revealed significant association of rs3914132 polymorphism with OTSC across different populations in different genetic models (Z = 6.707, p<0.0001). Statistical significance was evaluated through the *Z* and p values in the forest plot.

### Inheritance of a rare variant rs74503667

**Fig 3:**
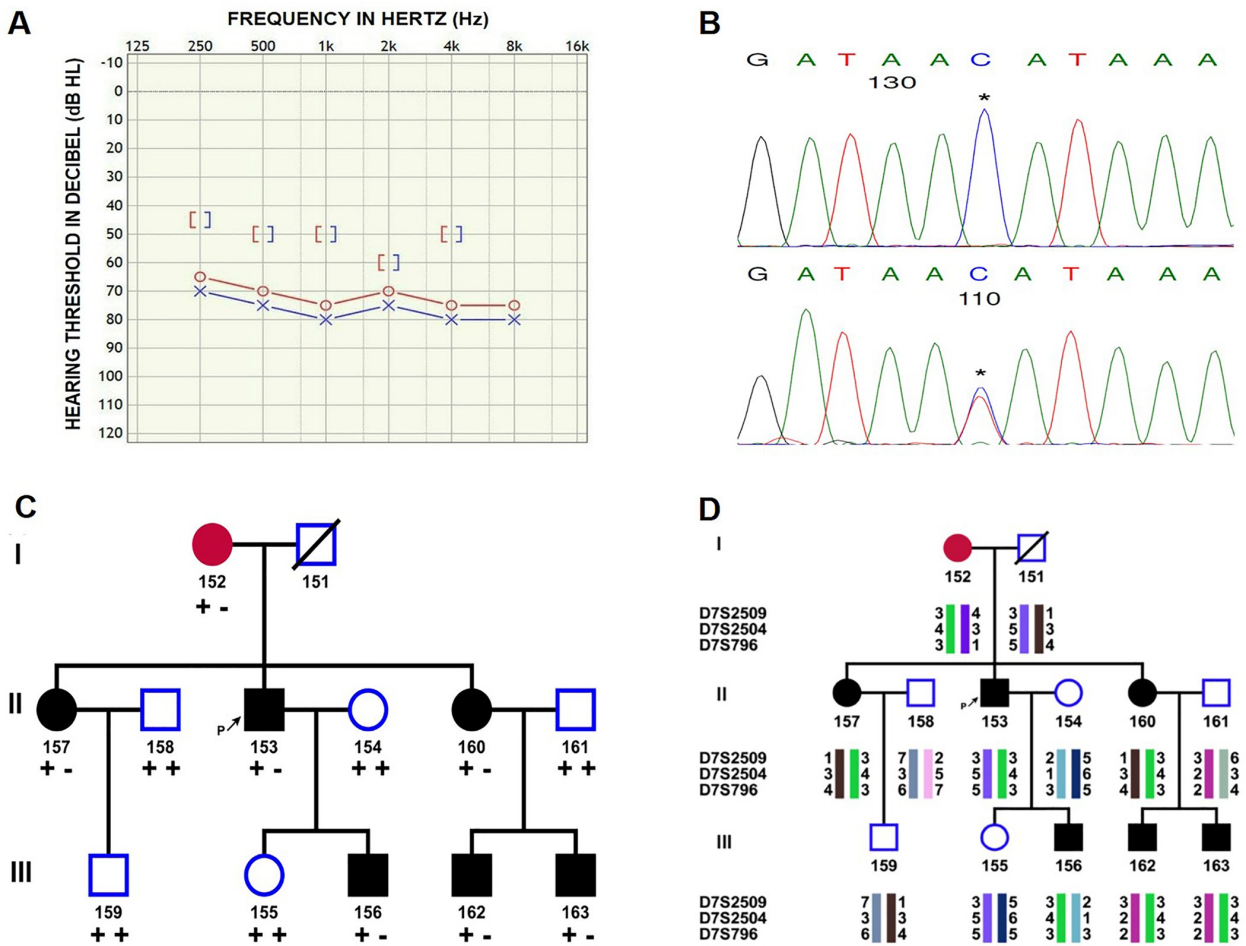
Transmission of disease associated alleles in OTSC family. (A)Typical audiogram of OTSC patient. On audiometric assessment, pure tone audiometry showed a bilateral severe mixed hearing loss in proband with an extent of severe hearing loss 71 dB in right ear and 75 dB in left ear. (B) Representative chromatograms showing the homozygous normal ‘C’ allele and heterozygous ‘CT’ genotype for rs74503667. (C) Inheritance of a rare variant rs74503667 in an OTSC family. Blackened squares and circles indicate affected males and females respectively. The **+ +** and **+—**symbols indicate the normal and heterozygous genotypes for the variant. Individual identity was represented by the numbers and arrow indicates proband. (D) Pedigree showing the transmission of a linked haplotype (green) with the disease at *RELN* locus (7q22-22.1). Circle with red indicates individual with uncertain status.

In our previous study, we identified a rare heterozygous variant rs74503667 at contig position 2923488 (NT_079596.2) in a familial OTSC case [16]. We extended the pedigree, and six subjects were diagnosed with OTSC spanning two generations. The proband (II: 153) was diagnosed with OTSC at the age of 30 years (Fig 3A). Genotyping analysis revealed the transmission of the heterozygous variant rs74503667 (Fig 3B). The proband, his sisters, son and two nephews were found to inherit this variation from his mother in an autosomal dominant mode (Fig 3C). We screened 254 sporadic cases and 262 controls together with 5 OTSC families consisting of 90 individuals; however, this variation was found to be inherited in only one family suggesting its role in monogenic form of OTSC.

### Familial OTSC is linked to *RELN* locus

We evaluated the linkage likelihood of *RELN* rs74503667 in the OTSC family. Based on the clinical and audiological parameters, the probands mother was considered as clinically uncertain. Linkage analysis in OTSC family was performed on a subset of 6 affected and 5 unaffected individuals (S1 Table). This family did not show linkage to known OTSC loci (S5 Table). However, this family identified the possible linked region at *RELN* locus on 7q22-22.1 in dominant mode and 90% penetrance level (Marker D7S796; two-point LOD score, 2.1059 at θ = 0.000) (Table 2). Multipoint linkage analysis using Gene Hunter program of easyLINKAGE assuming the same condition gave a maximum LOD score on chromosome 7q22-22.1 for marker D7S796 (Z_max_ = 2.1059). Haplotype analysis for *RELN* locus in this family showed the transmission of mutated haplotype in all the affected individuals through the probands mother (Fig 3D). The linkage, haplotype, and the transmission of rs74503667 in this family strongly suggest the pathogenic role of *RELN* in monogenic form of OTSC development.

**Table 2:** Two-point LOD score estimation for *RELN* loci in an OTSC family.

Locus	Marker	Two point LOD score at θ =				
		T0.000	T0.100	T0.200	T0.300	T0.400
*RELN*	D7S2509	1.5038	1.2301	0.9295	0.6081	0.2866
	D7S2504	1.8049	1.4396	1.0388	0.6183	0.2422
	D7S796	**2.1059**	1.7406	1.3378	0.9003	0.4449

The maximum two-point LOD score is marked in bold.

### *RELN* and *VLDLR* mRNA expression is reduced in otosclerotic stapes tissues

**Fig 4:**
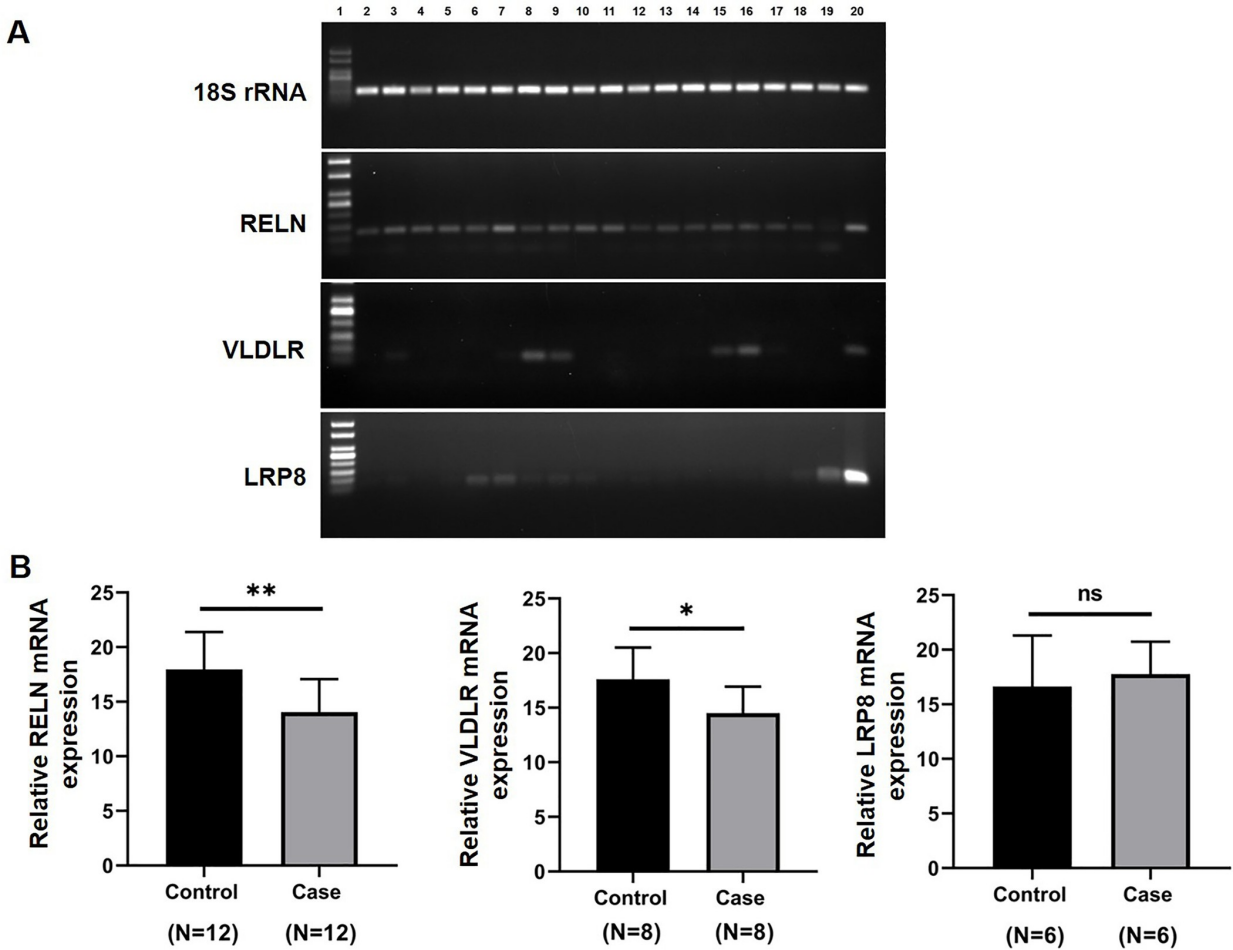
Comparison of mRNA expression of *RELN*, *VLDLR* and *LRP8* genes in stapes tissues of cases and controls. (A) Agarose gel electrophoresis of molecular weight marker (Lane 1, 100 bp for *18S rRNA* and *RELN*; 50 bp for *VLDLR* and *LRP8*), RT-PCR products of *18S rRNA* (151 bp), *RELN* (130 bp), *VLDLR* (88 bp), and *LRP8* (74 bp) in mRNA derived from stapes tissues of cases (Lane 2–10), controls (Lane 11–19) and incus bone (Lane 20). (B) Expression levels were quantified by RT-qPCR and normalized to *18S rRNA* expression. *RELN* expression was significantly lower in cases (p = 0.0068) compared to controls. *VLDLR* expression was found to be slightly lower in stapes tissues of cases (p = 0.0348) compared to controls. No remarkable change in *LRP8* expression was observed between cases (p = 0.6172) and controls. Results expressed as mean ± standard deviation of mean. *p<0.05, **p<0.01, ns = not significant.

The mRNA expression of *RELN* and its receptors *VLDLR* and *LRP8* were compared between otosclerotic and control stapes tissues using reverse transcriptase PCR and real-time PCR (Fig 4). RT-PCR analysis detected the expression of the reference gene *18S rRNA* in all specimens (OTSC stapes, N = 52; control stapes, N = 39; and incus bone, N = 3) with equal intensity ensuring the absence of RNA degradation (Table 3, Fig 4A).

**Table 3:** Expression of *RELN*, *VLDLR*, *LRP8* and *18S rRNA* genes in otosclerotic tissues and in tissue specific controls.

Specimens (N = 94)	*18S rRNA*	*RELN*	*VLDLR*	*LRP8*
Ankylotic Stapes tissues (N = 52)	52 (100%)	32 (61.5%)	17 (32.7%)	10 (19.2%)
Control Stapes tissues (N = 39)	39 (100%)	24 (61.5%)	13 (33.3%)	10 (25.6%)
Incus bone (N = 3)	03 (100%)	03 (100%)	03 (100%)	02 (66.6%)

Amongst the 52 otosclerotic stapes samples, 32 (61.5%) showed *RELN* expression with detectable levels and out of 39 control stapes, 24 (61.5%) showed *RELN* mRNA expression (p = 0.9999). *VLDLR* mRNA expression was detected in 32.7% cases and 33.3% controls (p = 0.9487). *LRP8* expression was detected in very limited number of stapes from cases (19.2%) and controls (25.6%) (p = 0.4649). *RELN* and *VLDLR* mRNA expressions were detected in all incus bones specimens, however, *LRP8* expression was detected in 2 out of 3 incus bones. This analysis revealed a limited sensitivity of RT-PCR for *RELN/VLDLR/LRP8* mRNA detection in stapes from cases and controls. The samples that were detected by RT-PCR were quantified for their levels of *RELN/VLDLR/LRP8* mRNA expressions. The data was normalised to the housekeeping gene *18S rRNA* and the relative fold change was calculated and validated using unpaired two-tailed Student’s t-test. The gene expression analysis revealed a significantly decreased level of *RELN* expression in otosclerotic stapes compared to controls (p = 0.0068). The *VLDLR* mRNA expression was also significantly reduced (p = 0.0348) in otosclerotic stapes compared to controls. However, *LRP8* expression was similar (p = 0.6172) in cases and control stapes (Fig 4B).

### Genetic variation in *RELN* impedes binding of TFs in mutated sequences

**Fig 5:**
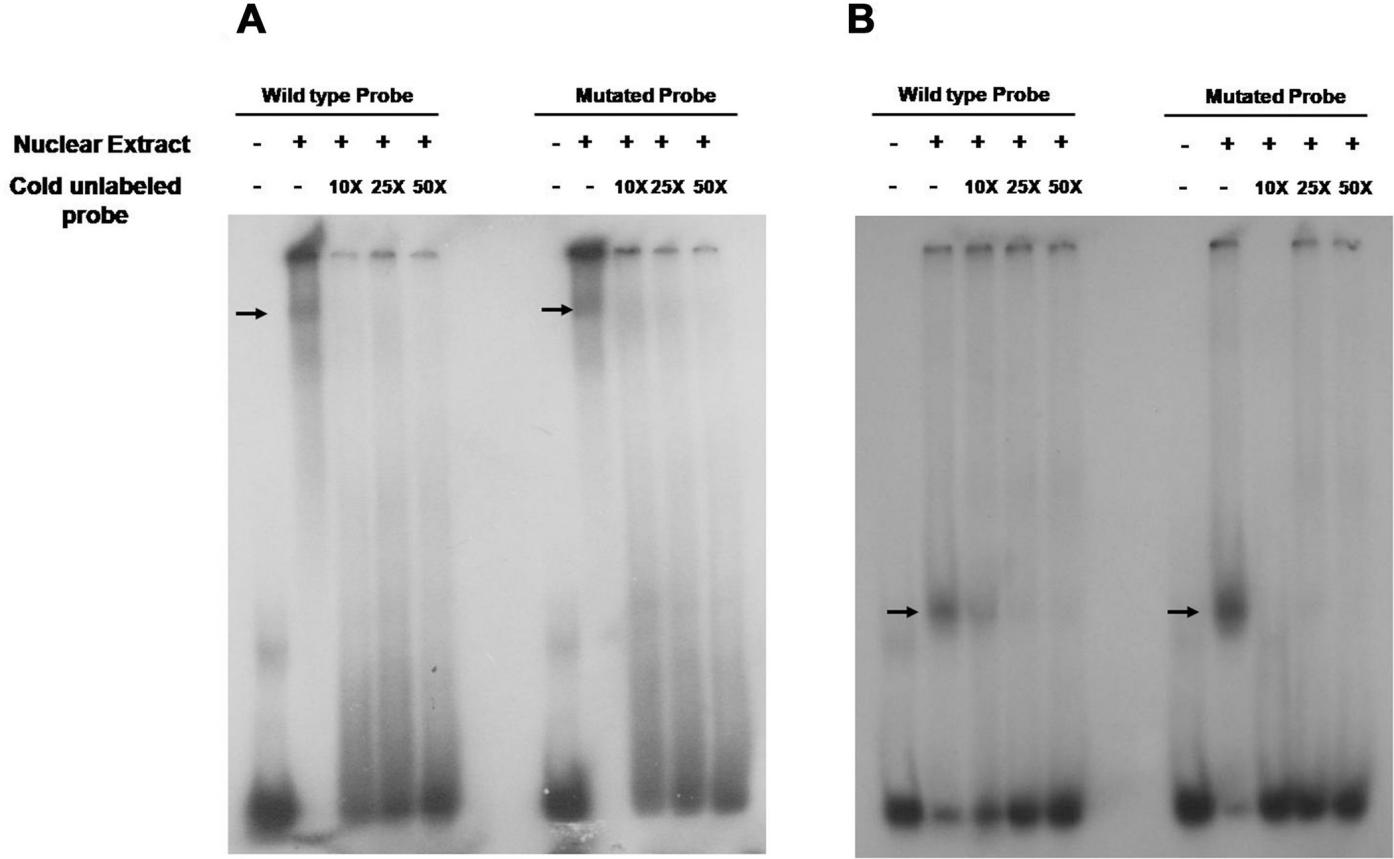
Electrophoretic mobility shift assay was performed on nuclear proteins extracted from human SH-SY5Y cells for *RELN* variants. (A) rs3914132 and (B) rs74503667 in intron 2 of *RELN* gene with either wild or mutant probes with (0X, 10X, 25X 50X) and without competitors. DNA-protein complex migration pattern was detected in both wild type and mutated probes for both the variations. The arrow indicates the DNA-protein binding at the sites of these variations.

To understand the functional potential of *RELN* gene variants, *in silico* analysis was performed using the ALGGEN PROMO program to predict the gain/loss of putative transcription factor binding sites (TFBSs). Ten base pairs of surrounding genomic DNA sequences were analysed using the optimized matrix similarity thresholds to predict gain and/or loss of putative TFBS. Analysing the *RELN* variant rs3914132 predicted the putative binding sites for GATA-1 in the mutated sequence. The rare variant rs74503667 in *RELN* predicted the altered binding of TATA element modulatory factor (TMF), TATA binding proteins (TBP) and Transcription factor II D (TFIID) in the mutated sequence. To investigate the binding affinity of the rare variant at rs74503667 and the SNP rs3914132 to nuclear protein, we performed EMSA with SH-SY5Y cells nuclear protein extracts with double stranded oligonucleotide probes containing either of the alleles. The experiment demonstrated the allelic difference in the intensity of bound DNA, with the nuclear protein (s) indicating the gain of possible transcription factors binding on altered sequence with ‘T’ allele of rs74503667 (p = 0.0010) as well as with the associated ‘C’ allele of rs3914132 (p = 0.0494) (Fig 5).

### Altered stapes bone morphology and reelin protein expression in OTSC

**Fig 6:**
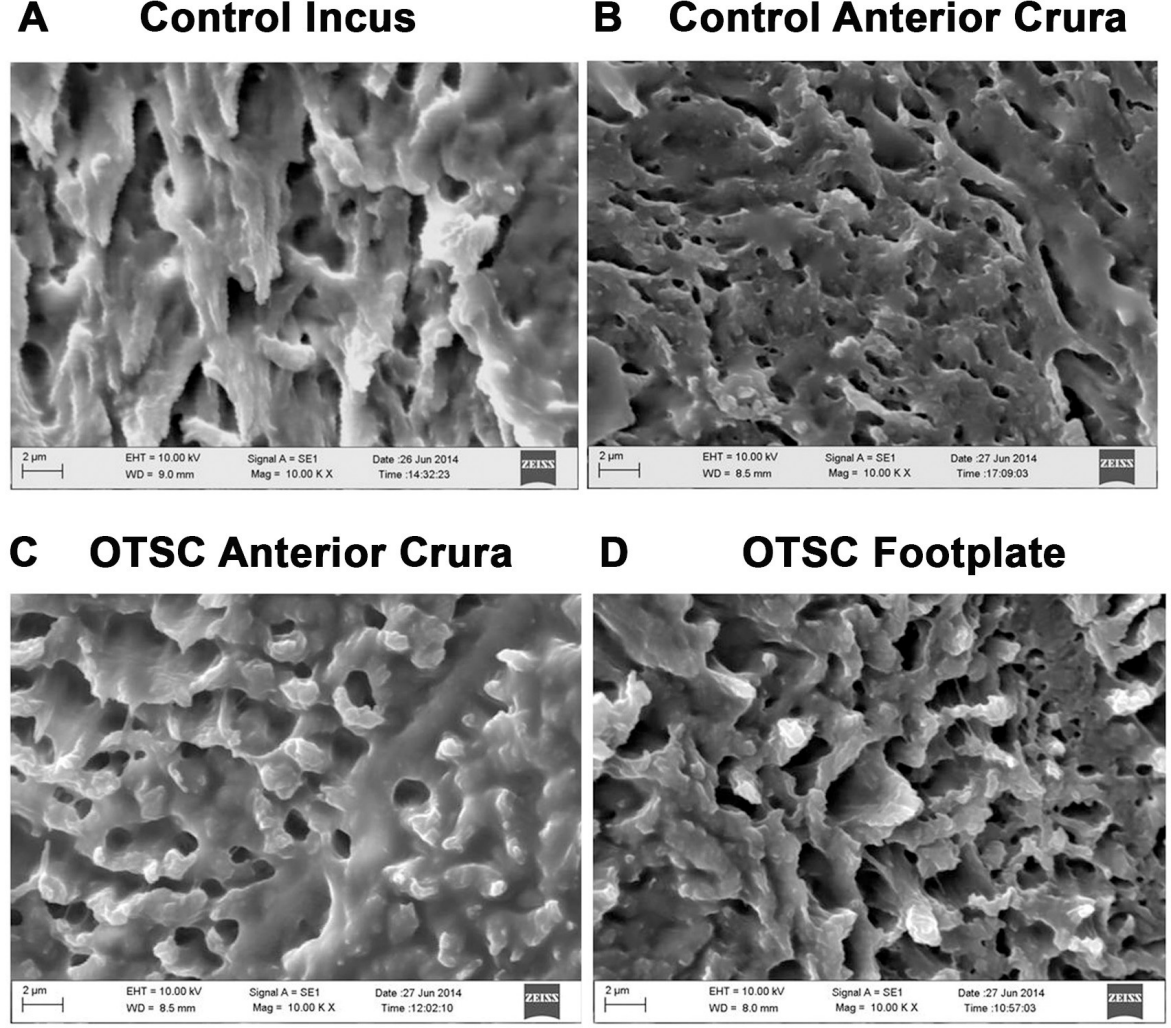
Scanning electron microscopy image of an otosclerotic stapes. The ultrastructure of the bone adopts a compact fibrillar morphological appearance in (A) incus bone from control, (B) anterior crura near otosclerotic zone in control stapes. Hollow honeycomb like structures were observed in (C) anterior crura near otosclerotic zone in patient’s stapes as well as in (D) stapedial footplate obtained from otosclerotic patient. Original magnification at 10000 times in a corresponding area of OTSC zone.

A comparative morphology analysis between case and control stapes was performed using scanning electron microscopy. Evaluation of anterior crura of control stapes showed a fibrillar structure, also present in otosclerotic free area of patient’s stapes (Fig 6). However, in patient’s stapes the otosclerotic zone of anterior crura and the footplate did not show any fibrillar structures. In spite, we observed numerous marks of multiple holes and honeycomb like porous structures in the otosclerotic zone of disease tissues compared to controls, which were less porous and more compact (S1 Fig). The incus bone also resembled structural pattern similar to control stapes (Fig 6A). This analysis showed that the morphology of the stapes is notably different in controls and OTSC patients.

**Fig 7:**
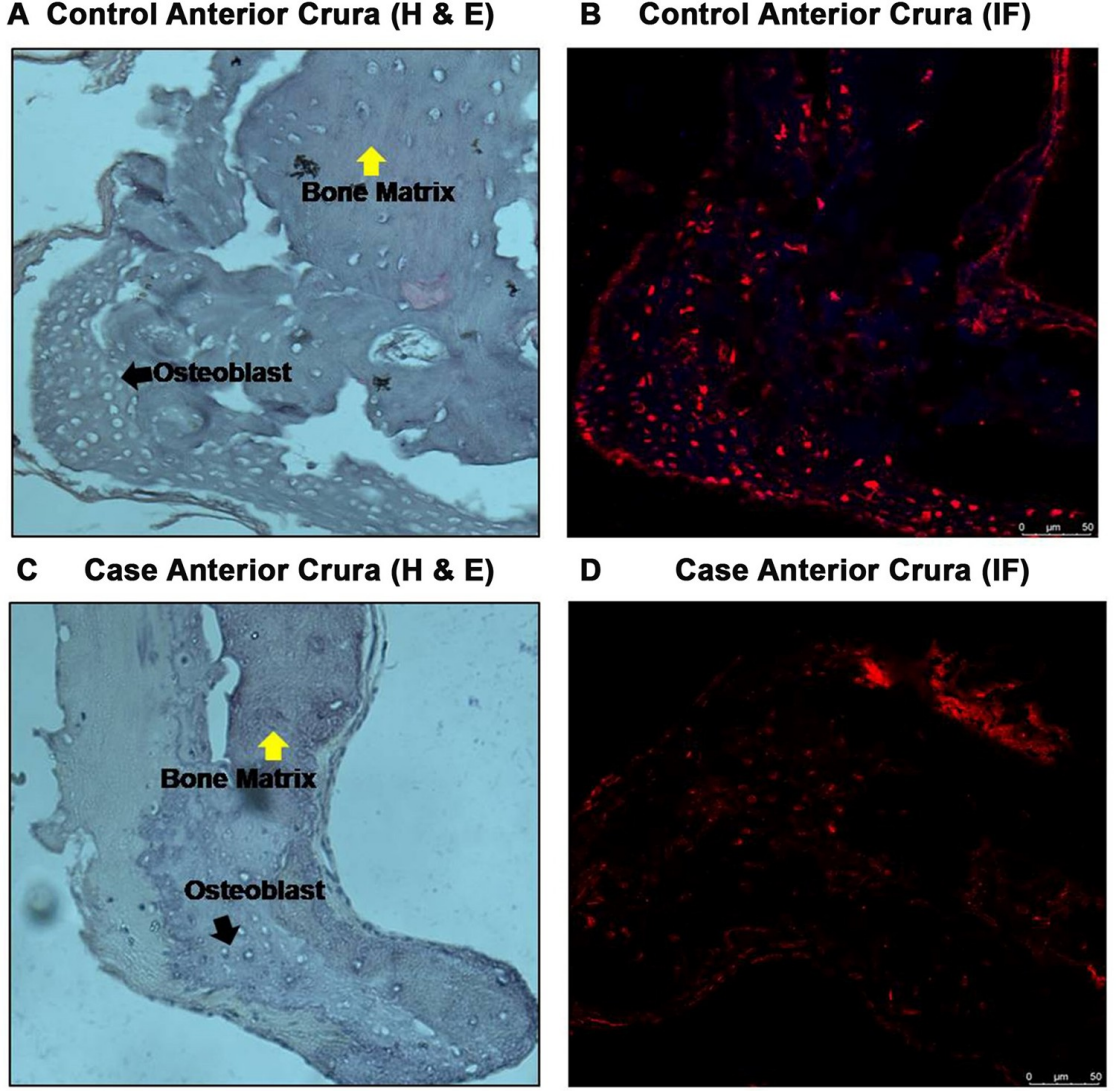
Histological analysis of stapes tissues. Haematoxylin-eosin (A, C) and immunofluorescence staining (B, D) showing the presence of bone cells and expression of reelin protein in stapes tissues from controls and cases. Immunofluorescence assay showed reduced reelin expression in otosclerotic tissues as compared to controls (scale bar = 50μm).

Subsequently, we investigated the reelin protein expression using immunofluorescence assay (IFA) in case and control stapes tissues. Histological examination of stapes tissues using haematoxylin and eosin staining showed osteoblast, osteoclast, and other bone cells like structures in both cases and controls (Fig 7A and 7C). IFA revealed a reduced reelin expression in OTSC tissues as compared to controls **(**Figs 7B, 7D and S2).

## Discussion

In the present study, we hypothesized that increasing the sample size will provide adequate power to detect the significance level. The low power (34%) used in our previous study [16] has been increased in the present study to reach 76% and we subsequently found that the association of the rs3914132 SNP with OTSC in Indian population is consistent with the previous GWAS study in European population [14]. The minor allele ‘C’ frequency for the SNP rs3914132 in patient and control group was found to be 0.09 and 0.15. The decreased minor allele frequency (MAF) in cases compared to controls indicates the protective effect towards OTSC development. In European population minor allele ‘C’ at rs3914132 in *RELN* gene acts as a protective allele and decreases the risk of OTSC by 1.54 times [14]. The association and estimated effect size for rs3914132 SNP with OTSC in this study was in accordance with the previous reports [1,15]. While comparing the allele counts between controls and cases, the odds ratio 1.756 (≅ 1.8) indicated that each ‘C’ allele reduces the OTSC susceptibility by 1.8 times in the studied population. Furthermore, the association of the same SNP with OTSC in our population confirmed that the association of rs3914132 in *RELN* gene with OTSC is real and *RELN* plays an important role in the etiopathogenesis of OTSC [1,15].

To confirm the genetic association of rs3914132 with OTSC, we performed a meta-analysis by combining the data from previous studies and the current study. The analysis confirmed the association of this SNP with OTSC across different ethnic populations with similar effect size, which further strengthen the evidence of genetic contribution of *RELN* in the development of OTSC (Fig 2). The functional role of this intronic SNP is not clear, however, it is possible that this SNP may influence the regulatory elements for other neighbouring genes which might have influence in causing the risk of OTSC development.

In our previous study, we had also identified a rare variation rs74503667 in a familial case of OTSC. Our sequencing result elucidated the transmission of rs74503667 variant in all the affected members of OTSC family (Fig 3C). Linkage analysis showed a suggestive linkage signal in this OTSC family to the *RELN* locus (LOD score 2.1059) (Table 2). Within the current study, eight candidate loci were tested therefore a LOD score > 2.0 is highly unlikely to occur by chance. According to Lander and Kruglayak, a LOD score above 1.9 correspond to suggestive linkage [48]. The same haplotype was found to be transmitted from proband’s mother to all the affected members (Fig 3D). This family did not show the evidence of linkage or transmission of haplotypes for any other loci identified previously for OTSC. Identification of linkage and transmission of rare novel variation in this family suggest the pathogenic role of *RELN* in OTSC.

The expression of *RELN* has been evidenced in human stapes footplate samples and human and mouse inner ear by real-time PCR and Western blot [14]. In the present study, determining the expression levels of the *RELN* together with two known receptors *VLDLR* and *LRP8* found a significant reduction in the expression of *RELN* and *VLDLR* in otosclerotic stapes, while, *LRP8* expression was similar in cases and control stapes. Studies have shown that osteocytes, the mechanosensing cells of the bone tissue, express higher levels of reelin than osteoblasts, the bone-forming cells [28]. The cell surface receptor VLDLR may transduce the reelin signaling to intracellular signaling molecules [49–51]. The decreased expression of *RELN* and *VLDLR* observed in otosclerotic stapes tissues compared with normal stapes can assist our understanding of the role of these genes in the causation of disease. Immunofluorescence study has also revealed that reelin immunoreactivity in the stapes of patients with otosclerosis was significantly lower than that of controls. Two previous studies reported conflicting results in detecting the RELN expression in stapes bone [14,52]. A recent study has shown the expression of *RELN* in the majority (62% of stapes bone tested) but not all human stapes tested [32]. Our results support the Mowat et al. findings which detected the RELN expression in 61.5% stapes bones tested in the present study. It has been postulated that expression of *RELN* in the stapes is interconnected with special features of the otic capsule [18]. Another study has shown the expression of *RELN* in adult rat appendicular and axial skeletons also [31]. However, lack of *RELN* expression in some of the otosclerotic stapes in this study is unclear, which could be influenced by other factors such as aberrant epigenetic modifications [28,53]. The possible cause of reduced expression of RELN in otosclerotic stapes could be due to a viral infection as demonstrated previously in different parts of mice brain [54–56]. Several reports have shown the implication of measles virus infection in the pathogenesis of OTSC, however, till date no correlation of measles virus infection on *RELN*/reelin expression in OTSC tissues was determined [57]. Other probable mechanism for reduced *RELN*/reelin expression could be due to disease associated polymorphism/variants identified in this study. Some reports have shown spontaneous mutations in *RELN* leads to lissencephaly and absence of serum reelin [21,58].

Functional analysis of *RELN* variants rs3914132 and rs74503667 by EMSA showed a notable difference in the formation of DNA-protein complex between the wild type and altered sequences. Difference in EMSA indicates that a protein or mixture of proteins is capable of binding to mutated DNA sequences for rs3914132 and rs74503667 which may have influence on the regulation of *RELN* or any other neighbouring gene located in same topologically associated domain. It may be possible that these intronic variants are in strong LD with the actual causative variants in the neighbouring candidate genes which might have a role in abnormal bone remodelling during otosclerosis development. The two proximally neighbouring genes can be *SLC26A5* and *ZKSCAN1*. Genetic variations in *SLC26A5* are associated with hearing impairment [14]. The *ZKSCAN1* was found to be differentially expressed in otosclerotic tissues [59], however, it is located 3,430,095 bp upstream to *RELN*.

Morphological analysis of human stapes tissues by SEM revealed aberrant bone structures due to OTSC with several hollow spheroids and honeycomb-like structures that were exclusively present in otosclerotic zone of the stapes. These aberrant structures were absent in control stapes as well as in incus bone fragments. In otosclerotic stapes, the bone is more porous which implies an increase in bone resorption activity due to the increased osteoclasts activity. In non-otosclerotic zone of the otosclerotic stapes, we found fibrillar structures similar to controls. The exact mechanism responsible for this abnormal bone anomaly in otosclerotic zone of stapes due to OTSC is not known. The same architectural pattern viz. honey comb, fibrillar compact and pitted was observed in the human stapes previously [60].

Based on the known biological function of reelin, it is difficult to correlate its role directly in bone metabolism, however, recent studies have shown the role of reelin signalling pathway as a prime role in limb development [61]. Reelin levels were also found to be increased in synovial fluid of patients with rheumatoid arthritis suggesting its role in musculoskeletal tissues [62]. Reports have shown the secretion of reelin in the inner ear and detection of its transcripts in human stapes footplate [14]. Some of the studies have shown the differential expression of reelin in osteocytes compared to osteoblasts [28]. Recently, Garshasbi et al. showed the role *RELN* variation with familial ankylosing spondylitis which further strengthen the role of RELN in bone remodeling [63]. We hypothesize from the current study and previous reports that the functional intronic variants rs3914132 and rs74503667 may be located in a regulatory region (enhancer/silencer) of *RELN* gene which might regulate the other neighbouring genes and may have role in controlling the bone remodeling. In addition, the reelin protein may contribute to mechanosensory adaptation mechanism of bone remodeling since it is detected with elevated expression in limb compared to skull bones. The exact mechanism by which reduced RELN expression is the causation of disease needs to be investigated. However, a recent study has investigated the association of ankylosis spondylitis (a bone disease) with RELN mutation which can alter the inflammatory and osteogenesis pathway mediated by reduced secretion of reelin [64]. In the current study, reduced *RELN* mRNA and reelin protein expressions in OTSC tissues together with association of *RELN* gene variants with OTSC in multiple populations provide deeper molecular understanding of *RELN* in disease susceptibility. Accumulating evidences from the studies suggests that signalling by reelin might be playing an important role in the pathogenies of otosclerosis.

Unfortunately, there are some limitations of this study. First, although the study contains a reasonable population size, the cohort is compromised with Indian-Asian individuals only and further ethnic populations like Africans or European populations might be included to further evidence the *RELN* variants in the risk of OTSC. In addition to further evaluate the exact functional role of reelin in OTSC disease, cell lines carrying the *RELN* variants should be included to provide evidence of its reduced RELN/reelin expression *in vitro*.

## Conclusions

In conclusion, this study provides an evidence that the rare variant rs74503667 in *RELN* has a large impact in the causation of monogenic form of OTSC and rs3914132 is associated with complex form of the disease. The quantified expression of *RELN*, *VLDLR* and *LRP8* in otosclerotic stapes may assist in understanding the mechanisms of reelin signalling in abnormal bone growth. However, more studies are needed in order to elucidate the exact role of reelin in abnormal bone growth around the stapes.

## Supporting information

S1 Fig Scanning electron microscopy image of a normal and otosclerotic stapes. The ultrastructure of the bones adopts a fibrillar morphological appearance in (A) Anterior Crura of control stapes bone and (B) Anterior Crura of patient’s stapes bone (original magnification X 5000) in a corresponding area to otosclerosis–free zone.
(TIF)

S2 Fig Immunofluorescence staining to determine RELN expression in stapes tissue samples. (A) Control stapes. (B) Otosclerosis stapes. (Scale bar = 50 μm). Immunofluorescence assay showed reduced reelin expression in otosclerotic tissues as compared to controls.
(TIF)

S1 Table Phenotypic and clinical description of subjects in otosclerosis family. M: Male; F: Female, BCHL = Bilateral conductive hearing loss; BMHL = Bilateral mixed hearing loss.
(DOCX)

S2 Table Microsatellite markers for linkage analysis of reported loci. Chr = chromosome, ASR = alleles size range, FAM = 6-arboxyfluorescein dye, HEX = 6-Hexachloro-fluorescein dye.
(DOCX)

S3 Table Semi quantitative PCR and real-time PCR primers used for gene expression analysis. F, forward primer; R, reverse primer; bp, base pair.
(DOCX)

S4 Table Characteristics of the eligible studies used for meta-analysis. Notes: *comprised European population from the genome wide association study (DG: Discovery group, RS1: Replication set 1, RS2: Replication Set 2). **comprised four cohorts: German, Italian, Swiss and Romanian.
(DOCX)

S5 Table Two-point LOD score estimation for known OTSC loci in a family. (DOCX)

S1 Raw images (PDF)

## References

[1] Khalfallah A, Schrauwen I, Mnaja M, Fransen E, Lahmar I, Ealy M, et al. Genetic variants in RELN are associated with otosclerosis in a non-European population from Tunisia. Ann Hum Genet. 2010;74(5):399–405. Epub 2010/07/21. doi: 10.1111/j.1469-1809.2010.00595.x . 20642811

[2] Kapur YP, Patt AJ. Otosclerosis in South India. Acta Oto-Laryngologica. 1966;61(1–6):353–60. doi: 10.3109/00016486609127071 5956101

[3] Fowler EP. Otosclerosis in identical twins. A study of 40 pairs. Arch Otolaryngol. 1966;83(4):324–8. Epub 1966/04/01. doi: 10.1001/archotol.1966.00760020326006 . 5907023

[4] Babcock TA, Liu XZ. Otosclerosis: From Genetics to Molecular Biology. Otolaryngol Clin North Am. 2018;51(2):305–18. Epub 2018/03/06. doi: 10.1016/j.otc.2017.11.002 . 29502723

[5] Tavernier LJM, Fransen E, Valgaeren H, Van Camp G. Genetics of otosclerosis: finally catching up with other complex traits? Hum Genet. 2021. Epub 2021/09/10. doi: 10.1007/s00439-021-02357-1 34498117

[6] Schrauwen I, Khalfallah A, Ealy M, Fransen E, Claes C, Huber A, et al. COL1A1 association and otosclerosis: a meta-analysis. Am J Med Genet A. 2012;158A(5):1066–70. Epub 2012/04/11. doi: 10.1002/ajmg.a.35276 . 22489040

[7] Hansdah K, Singh N, Bouzid A, Priyadarshi S, Ray CS, Desai A, et al. Evaluation of the Genetic Association and mRNA Expression of the COL1A1, BMP2, and BMP4 Genes in the Development of Otosclerosis. Genet Test Mol Biomarkers. 2020;24(6):343–51. Epub 2020/05/08. doi: 10.1089/gtmb.2019.0235 . 32379989

[8] Thys M, Schrauwen I, Vanderstraeten K, Janssens K, Dieltjens N, Van Den Bogaert K, et al. The coding polymorphism T263I in TGF-beta1 is associated with otosclerosis in two independent populations. Hum Mol Genet. 2007;16(17):2021–30. Epub 2007/06/26. doi: 10.1093/hmg/ddm150 . 17588962

[9] Priyadarshi S, Hansdah K, Ray CS, Biswal NC, Ramchander PV. Otosclerosis Associated with a De Novo Mutation -832G > A in the TGFB1 Gene Promoter Causing a Decreased Expression Level. Sci Rep. 2016;6:29572. Epub 2016/07/13. doi: 10.1038/srep29572 ; PubMed Central PMCID: PMC4941736. 27404893 PMC4941736

[10] Schrauwen I, Thys M, Vanderstraeten K, Fransen E, Dieltjens N, Huyghe JR, et al. Association of bone morphogenetic proteins with otosclerosis. J Bone Miner Res. 2008;23(4):507–16. Epub 2007/11/21. doi: 10.1359/jbmr.071112 ; PubMed Central PMCID: PMC2669162. 18021008 PMC2669162

[11] Priyadarshi S, Ray CS, Biswal NC, Nayak SR, Panda KC, Desai A, et al. Genetic association and altered gene expression of osteoprotegerin in otosclerosis patients. Ann Hum Genet. 2015;79(4):225–37. Epub 2015/05/23. doi: 10.1111/ahg.12118 . 25998045

[12] Bouzid A, Tekari A, Jbeli F, Chakroun A, Hansdah K, Souissi A, et al. Osteoprotegerin gene polymorphisms and otosclerosis: an additional genetic association study, multilocus interaction and meta-analysis. BMC Med Genet. 2020;21(1):122. Epub 2020/06/05. doi: 10.1186/s12881-020-01036-8 ; PubMed Central PMCID: PMC7268516. 32493243 PMC7268516

[13] Hojland AT, Tavernier LJM, Schrauwen I, Sommen M, Topsakal V, Schatteman I, et al. A wide range of protective and predisposing variants in aggrecan influence the susceptibility for otosclerosis. Hum Genet. 2021. Epub 2021/08/20. doi: 10.1007/s00439-021-02334-8 . 34410490

[14] Schrauwen I, Ealy M, Huentelman MJ, Thys M, Homer N, Vanderstraeten K, et al. A genome-wide analysis identifies genetic variants in the RELN gene associated with otosclerosis. American journal of human genetics. 2009;84(3):328–38. Epub 2009/02/24. doi: 10.1016/j.ajhg.2009.01.023 ; PubMed Central PMCID: PMC2667982. 19230858 PMC2667982

[15] Schrauwen I, Ealy M, Fransen E, Vanderstraeten K, Thys M, Meyer NC, et al. Genetic variants in the RELN gene are associated with otosclerosis in multiple European populations. Hum Genet. 2010;127(2):155–62. Epub 2009/10/23. doi: 10.1007/s00439-009-0754-2 . 19847460

[16] Priyadarshi S, Panda KC, Panda AK, Ramchander PV. Lack of association between SNP rs3914132 of the RELN gene and otosclerosis in India. Genet Mol Res. 2010;9(3):1914–20. Epub 2010/10/01. doi: 10.4238/vol9-3gmr890 . 20882487

[17] Sommen M, Van Camp G, Liktor B, Csomor P, Fransen E, Sziklai I, et al. Genetic association analysis in a clinically and histologically confirmed otosclerosis population confirms association with the TGFB1 gene but suggests an association of the RELN gene with a clinically indistinguishable otosclerosis-like phenotype. Otol Neurotol. 2014;35(6):1058–64. Epub 2014/03/20. doi: 10.1097/MAO.0000000000000334 . 24643032

[18] Nielsen KB, Sondergaard A, Johansen MG, Schauser K, Vejlsted M, Nielsen AL, et al. Reelin expression during embryonic development of the pig brain. BMC neuroscience. 2010;11:75. Epub 2010/06/17. doi: 10.1186/1471-2202-11-75 ; PubMed Central PMCID: PMC2895594. 20550682 PMC2895594

[19] Roberts RC, Xu L, Roche JK, Kirkpatrick B. Ultrastructural localization of reelin in the cortex in post-mortem human brain. The Journal of comparative neurology. 2005;482(3):294–308. Epub 2005/02/04. doi: 10.1002/cne.20408 . 15690491

[20] Armstrong NC, Anderson RC, McDermott KW. Reelin: Diverse roles in central nervous system development, health and disease. Int J Biochem Cell Biol. 2019;112:72–5. Epub 2019/04/26. doi: 10.1016/j.biocel.2019.04.009 . 31022460

[21] Hong SE, Shugart YY, Huang DT, Shahwan SA, Grant PE, Hourihane JO, et al. Autosomal recessive lissencephaly with cerebellar hypoplasia is associated with human RELN mutations. Nat Genet. 2000;26(1):93–6. Epub 2000/09/06. doi: 10.1038/79246 . 10973257

[22] Fatemi SH, Snow AV, Stary JM, Araghi-Niknam M, Reutiman TJ, Lee S, et al. Reelin signaling is impaired in autism. Biological psychiatry. 2005;57(7):777–87. doi: 10.1016/j.biopsych.2004.12.018 15820235

[23] Förster E, Bock HH, Herz J, Chai X, Frotscher M, Zhao S. Emerging topics in Reelin function. European Journal of Neuroscience. 2010;31(9):1511–8. doi: 10.1111/j.1460-9568.2010.07222.x 20525064 PMC2942760

[24] Goes FS, Willour VL, Zandi PP, Belmonte PL, MacKinnon DF, Mondimore FM, et al. Sex-specific association of the Reelin gene with bipolar disorder. American journal of medical genetics Part B, Neuropsychiatric genetics. 2010;153B(2):549–53. Epub 2009/08/20. doi: 10.1002/ajmg.b.31018 ; PubMed Central PMCID: PMC3032172. 19691043 PMC3032172

[25] Shifman S, Johannesson M, Bronstein M, Chen SX, Collier DA, Craddock NJ, et al. Genome-wide association identifies a common variant in the reelin gene that increases the risk of schizophrenia only in women. PLoS genetics. 2008;4(2):e28. doi: 10.1371/journal.pgen.0040028 18282107 PMC2242812

[26] Skaar D, Shao Y, Haines J, Stenger J, Jaworski J, Martin E, et al. Analysis of the RELN gene as a genetic risk factor for autism. Molecular psychiatry. 2005;10(6):563–71. doi: 10.1038/sj.mp.4001614 15558079

[27] Dou A, Zhang Y, Wang Y, Liu X, Guo Y. Reelin depletion alleviates multiple myeloma bone disease by promoting osteogenesis and inhibiting osteolysis. Cell death discovery. 2021;7(1):219. Epub 2021/08/27. doi: 10.1038/s41420-021-00608-8 ; PubMed Central PMCID: PMC8387418. 34433809 PMC8387418

[28] Paic F, Igwe JC, Nori R, Kronenberg MS, Franceschetti T, Harrington P, et al. Identification of differentially expressed genes between osteoblasts and osteocytes. Bone. 2009;45(4):682–92. Epub 2009/06/23. doi: 10.1016/j.bone.2009.06.010 ; PubMed Central PMCID: PMC2731004. 19539797 PMC2731004

[29] Ma Y, Li X, Fu J, Li Y, Gao L, Yang L, et al. Acetylcholine affects osteocytic MLO-Y4 cells via acetylcholine receptors. Molecular and cellular endocrinology. 2014;384(1–2):155–64. Epub 2014/02/11. doi: 10.1016/j.mce.2014.01.021 . 24508663

[30] Khialeeva E, Carpenter EM. Nonneuronal roles for the reelin signaling pathway. Developmental dynamics. 2017;246(4):217–26. Epub 2016/10/16. doi: 10.1002/dvdy.24462 . 27739126

[31] Rawlinson SC, McKay IJ, Ghuman M, Wellmann C, Ryan P, Prajaneh S, et al. Adult rat bones maintain distinct regionalized expression of markers associated with their development. PLoS One. 2009;4(12):e8358. Epub 2009/12/23. doi: 10.1371/journal.pone.0008358 ; PubMed Central PMCID: PMC2792039. 20027296 PMC2792039

[32] Mowat AJ, Crompton M, Ziff JL, Aldren CP, Lavy JA, Saeed SR, et al. Evidence of distinct RELN and TGFB1 genetic associations in familial and non-familial otosclerosis in a British population. Hum Genet. 2018;137(5):357–63. Epub 2018/05/08. doi: 10.1007/s00439-018-1889-9 ; PubMed Central PMCID: PMC5973954. 29728750 PMC5973954

[33] Lahiri DK, Nurnberger JI Jr. A rapid non-enzymatic method for the preparation of HMW DNA from blood for RFLP studies. Nucleic Acids Res. 1991;19(19):5444. Epub 1991/10/21. doi: 10.1093/nar/19.19.5444 PubMed Central PMCID: PMC328920. 1681511 PMC328920

[34] Borenstein M, Hedges L, Higgins J, Rothstein H. Comprehensive meta-analysis version 3. Englewood NJ: Biostat. Inc; 2005.

[35] Melsen WG, Bootsma MC, Rovers MM, Bonten MJ. The effects of clinical and statistical heterogeneity on the predictive values of results from meta-analyses. Clinical microbiology and infection. 2014;20(2):123–9. Epub 2013/12/11. doi: 10.1111/1469-0691.12494 . 24320992

[36] Borenstein M, Hedges LV, Higgins JP, Rothstein HR. A basic introduction to fixed-effect and random-effects models for meta-analysis. Research synthesis methods. 2010;1(2):97–111. Epub 2010/04/01. doi: 10.1002/jrsm.12 . 26061376

[37] Begg CB, Mazumdar M. Operating characteristics of a rank correlation test for publication bias. Biometrics. 1994;50(4):1088–101. Epub 1994/12/01. . 7786990

[38] Egger M, Davey Smith G, Schneider M, Minder C. Bias in meta-analysis detected by a simple, graphical test. Bmj. 1997;315(7109):629–34. Epub 1997/10/06. doi: 10.1136/bmj.315.7109.629 ; PubMed Central PMCID: PMC2127453. 9310563 PMC2127453

[39] Mathur MB, VanderWeele TJ. Sensitivity analysis for publication bias in meta-analyses. Journal of the Royal Statistical Society Series C, Applied statistics. 2020;69(5):1091–119. Epub 2020/11/03. doi: 10.1111/rssc.12440 ; PubMed Central PMCID: PMC7590147. 33132447 PMC7590147

[40] Hoffmann K, Lindner TH. easyLINKAGE-Plus—automated linkage analyses using large-scale SNP data. Bioinformatics. 2005;21(17):3565–7. Epub 2005/07/15. doi: 10.1093/bioinformatics/bti571 . 16014370

[41] Thiele H, Nurnberg P. HaploPainter: a tool for drawing pedigrees with complex haplotypes. Bioinformatics. 2005;21(8):1730–2. Epub 2004/09/21. doi: 10.1093/bioinformatics/bth488 . 15377505

[42] Priyadarshi S, Ray CS, Panda KC, Desai A, Nayak SR, Biswal NC, et al. Genetic association and gene expression profiles of TGFB1 and the contribution of TGFB1 to otosclerosis susceptibility. J Bone Miner Res. 2013;28(12):2490–7. Epub 2013/05/25. doi: 10.1002/jbmr.1991 . 23703862

[43] Hellman LM, Fried MG. Electrophoretic mobility shift assay (EMSA) for detecting protein-nucleic acid interactions. Nature protocols. 2007;2(8):1849–61. Epub 2007/08/19. doi: 10.1038/nprot.2007.249 ; PubMed Central PMCID: PMC2757439. 17703195 PMC2757439

[44] Wiatr A, Skladzien J, Swiezy K, Wiatr M. A Biochemical Analysis of the Stapes. Medical science monitor: international medical journal of experimental and clinical research. 2019;25:2679–86. Epub 2019/04/13. doi: 10.12659/MSM.913635 ; PubMed Central PMCID: PMC6475125. 30975972 PMC6475125

[45] Bataille F, Troppmann S, Klebl F, Rogler G, Stoelcker B, Hofstadter F, et al. Multiparameter immunofluorescence on paraffin-embedded tissue sections. Applied immunohistochemistry & molecular morphology: AIMM. 2006;14(2):225–8. Epub 2006/06/21. doi: 10.1097/01.pai.0000162009.31931.10 . 16785795

[46] Purcell S, Cherny SS, Sham PC. Genetic Power Calculator: design of linkage and association genetic mapping studies of complex traits. Bioinformatics. 2003;19(1):149–50. Epub 2002/12/25. doi: 10.1093/bioinformatics/19.1.149 . 12499305

[47] Rodriguez S, Gaunt TR, Day IN. Hardy-Weinberg equilibrium testing of biological ascertainment for Mendelian randomization studies. American journal of epidemiology. 2009;169(4):505–14. Epub 2009/01/08. doi: 10.1093/aje/kwn359 ; PubMed Central PMCID: PMC2640163. 19126586 PMC2640163

[48] Lander E, Kruglyak L. Genetic dissection of complex traits: guidelines for interpreting and reporting linkage results. Nat Genet. 1995;11(3):241–7. Epub 1995/11/01. doi: 10.1038/ng1195-241 . 7581446

[49] Herz J, Chen Y. Reelin, lipoprotein receptors and synaptic plasticity. Nature reviews Neuroscience. 2006;7(11):850–9. Epub 2006/10/21. doi: 10.1038/nrn2009 . 17053810

[50] Hiesberger T, Trommsdorff M, Howell BW, Goffinet A, Mumby MC, Cooper JA, et al. Direct binding of Reelin to VLDL receptor and ApoE receptor 2 induces tyrosine phosphorylation of disabled-1 and modulates tau phosphorylation. Neuron. 1999;24(2):481–9. Epub 1999/11/26. doi: 10.1016/s0896-6273(00)80861-2 . 10571241

[51] D’Arcangelo G, Homayouni R, Keshvara L, Rice DS, Sheldon M, Curran T. Reelin is a ligand for lipoprotein receptors. Neuron. 1999;24(2):471–9. doi: 10.1016/s0896-6273(00)80860-0 10571240

[52] Csomor P, Sziklai I, Karosi T. Controversies in RELN/reelin expression in otosclerosis. Eur Arch Otorhinolaryngol. 2012;269(2):431–40. Epub 2011/06/02. doi: 10.1007/s00405-011-1653-4 . 21630058

[53] Abdolmaleky HM, Smith CL, Faraone SV, Shafa R, Stone W, Glatt SJ, et al. Methylomics in psychiatry: Modulation of gene-environment interactions may be through DNA methylation. American journal of medical genetics Part B, Neuropsychiatric genetics. 2004;127B(1):51–9. Epub 2004/04/27. doi: 10.1002/ajmg.b.20142 . 15108180

[54] Fatemi SH, Emamian ES, Kist D, Sidwell RW, Nakajima K, Akhter P, et al. Defective corticogenesis and reduction in Reelin immunoreactivity in cortex and hippocampus of prenatally infected neonatal mice. Mol Psychiatry. 1999;4(2):145–54. Epub 1999/04/20. doi: 10.1038/sj.mp.4000520 . 10208446

[55] Meyer U, Nyffeler M, Engler A, Urwyler A, Schedlowski M, Knuesel I, et al. The time of prenatal immune challenge determines the specificity of inflammation-mediated brain and behavioral pathology. J Neurosci. 2006;26(18):4752–62. Epub 2006/05/05. doi: 10.1523/JNEUROSCI.0099-06.2006 ; PubMed Central PMCID: PMC6674174. 16672647 PMC6674174

[56] Meyer U, Nyffeler M, Yee BK, Knuesel I, Feldon J. Adult brain and behavioral pathological markers of prenatal immune challenge during early/middle and late fetal development in mice. Brain Behav Immun. 2008;22(4):469–86. Epub 2007/11/21. doi: 10.1016/j.bbi.2007.09.012 . 18023140

[57] Karosi T, Szalmas A, Csomor P, Konya J, Petko M, Sziklai I. Disease-associated novel CD46 splicing variants and pathologic bone remodeling in otosclerosis. Laryngoscope. 2008;118(9):1669–76. Epub 2008/08/05. doi: 10.1097/MLG.0b013e31817c133d . 18677279

[58] Chang BS, Duzcan F, Kim S, Cinbis M, Aggarwal A, Apse KA, et al. The role of RELN in lissencephaly and neuropsychiatric disease. American journal of medical genetics Part B, Neuropsychiatric genetics. 2007;144B(1):58–63. Epub 2006/09/08. doi: 10.1002/ajmg.b.30392 . 16958033

[59] Ealy M, Chen W, Ryu GY, Yoon JG, Welling DB, Hansen M, et al. Gene expression analysis of human otosclerotic stapedial footplates. Hear Res. 2008;240(1–2):80–6. Epub 2008/04/24. doi: 10.1016/j.heares.2008.03.001 ; PubMed Central PMCID: PMC2442649. 18430532 PMC2442649

[60] Graham MD, House HP. Human stapes crura: surface bone architecture: scanning electron microscopic findings. Laryngoscope. 1976;86(7):1008–14. Epub 1976/07/01. doi: 10.1288/00005537-197607000-00016 . 933681

[61] Diaz-Mendoza MJ, Lorda-Diez CI, Montero JA, Garcia-Porrero JA, Hurle JM. Reelin/DAB-1 signaling in the embryonic limb regulates the chondrogenic differentiation of digit mesodermal progenitors. J Cell Physiol. 2014;229(10):1397–404. Epub 2014/02/13. doi: 10.1002/jcp.24576 . 24519818

[62] Magnani A, Pattacini L, Boiardi L, Casali B, Salvarani C. Reelin levels are increased in synovial fluid of patients with rheumatoid arthritis. Clin Exp Rheumatol. 2010;28(4):546–8. Epub 2010/07/28. . 20659411

[63] Garshasbi M, Mahmoudi M, Razmara E, Vojdanian M, Aslani S, Farhadi E, et al. Identification of RELN variant p.(Ser2486Gly) in an Iranian family with ankylosing spondylitis; the first association of RELN and AS. Eur J Hum Genet. 2020;28(6):754–62. Epub 2020/02/01. doi: 10.1038/s41431-020-0573-4 ; PubMed Central PMCID: PMC7253431. 32001840 PMC7253431

[64] Esmaeilzadeh-Gharehdaghi E, Razmara E, Bitaraf A, Jamshidi A, Mahmoudi M, Garshasbi M. Functional Analysis of RELN S2486G Mutation and its Contribution to Pathogenesis of Ankylosing Spondylitis. Archives of Iranian medicine. 2020;23(10):688–96. Epub 2020/10/28. doi: 10.34172/aim.2020.87 . 33107310

