# Supplementary figures and images for: The risks of RELN polymorphisms and its expression in the development of otosclerosis
Source: PLoS One. 2022 Jun 3;17(6):e0269558. doi: 10.1371/journal.pone.0269558 (PMC9165908; doi:10.1371/journal.pone.0269558)

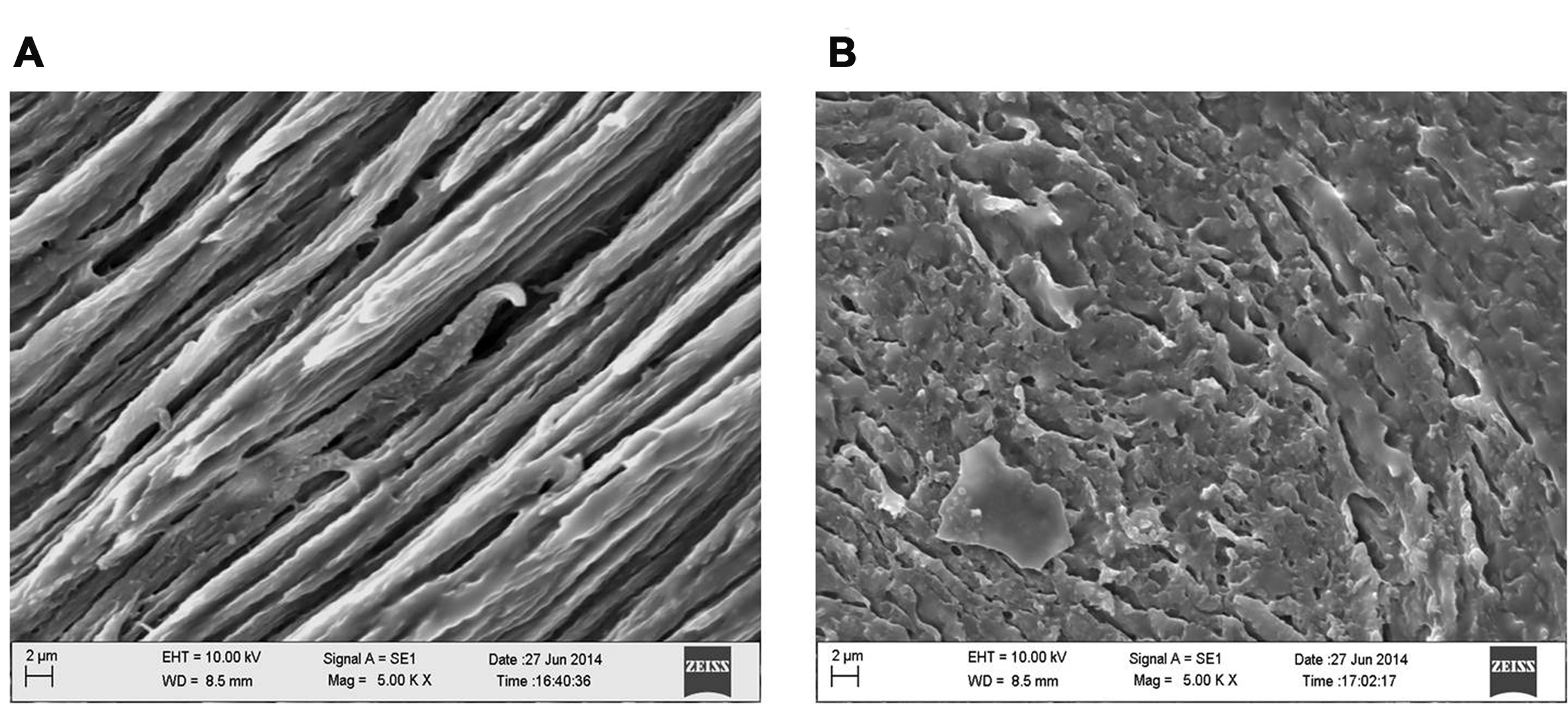

Supplement: S1 Fig — The ultrastructure of the bones adopts a fibrillar morphological appearance in (A) Anterior Crura of control stapes bone and (B) Anterior Crura of patient’s stapes bone (original magnification X 5000) in a corresponding area to otosclerosis–free zone. (TIF) [file pone.0269558.s001.tif]

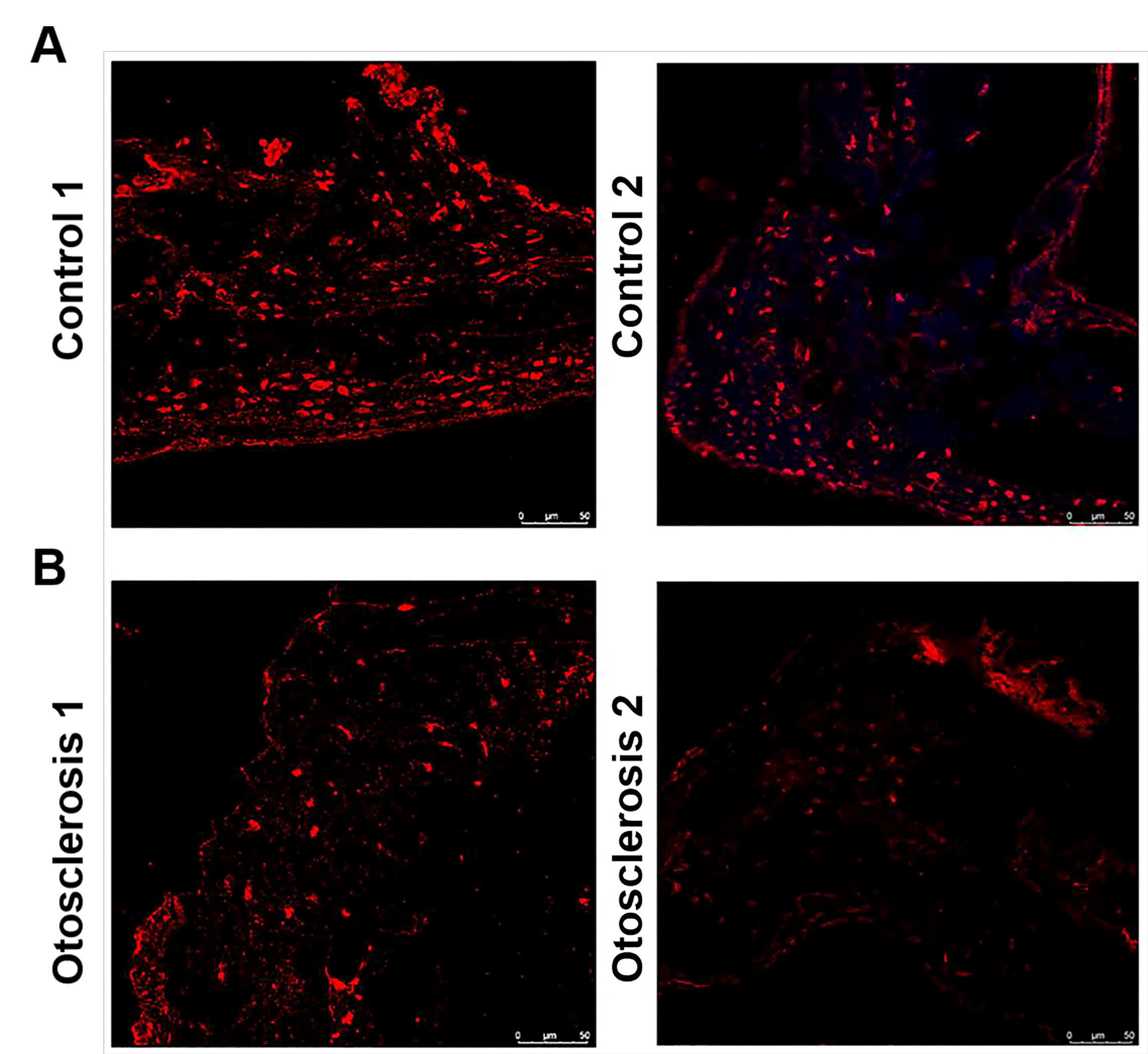

Supplement: S2 Fig — (A) Control stapes. (B) Otosclerosis stapes. (Scale bar = 50 μm). Immunofluorescence assay showed reduced reelin expression in otosclerotic tissues as compared to controls. (TIF) [file pone.0269558.s002.tif]
