# Supplementary material for: The risks of RELN polymorphisms and its expression in the development of otosclerosis
Source: PLoS One. 2022 Jun 3;17(6):e0269558. doi: 10.1371/journal.pone.0269558 (PMC9165908; doi:10.1371/journal.pone.0269558)
Supplement: S1 Table — M: Male; F: Female, BCHL = Bilateral conductive hearing loss; BMHL = Bilateral mixed hearing loss. (DOCX) [file pone.0269558.s003.docx]

# S1 Table. Phenotypic and clinical description of subjects in otosclerosis family

| **S. No.** | **Family ID** | **Subject**  **ID** | **Gender** | **Age** | **Age at**  **Onset** | **Diagnosis** | **Stapedectomy** |
| --- | --- | --- | --- | --- | --- | --- | --- |
| 1 | I | 151 | M |  |  | Deceased |  |
| 2 | I | 152 | F | 75 |  | Unknown |  |
| 3 | I | 153 | M | 45 | 30 | BMHL | Yes |
| 4 | I | 154 | F | 40 |  | Normal |  |
| 5 | I | 155 | F | 20 |  | Normal |  |
| 6 | I | 156 | M | 18 | 15 | BCHL |  |
| 7 | I | 157 | F | 50 | 35 | BMHL | Yes |
| 8 | I | 158 | M | 55 |  | Normal |  |
| 9 | I | 159 | M | 25 |  | Normal |  |
| 10 | I | 160 | F | 48 | 32 | BCHL |  |
| 11 | I | 161 | M | 53 |  | Normal |  |
| 12 | I | 162 | M | 30 | 22 | BCHL |  |
| 13 | I | 163 | M | 26 | 20 | BCHL |  |

M: Male; F: Female, BCHL = Bilateral conductive hearing loss; BMHL = Bilateral mixed hearing loss
