# Supplementary material for: The risks of RELN polymorphisms and its expression in the development of otosclerosis
Source: PLoS One. 2022 Jun 3;17(6):e0269558. doi: 10.1371/journal.pone.0269558 (PMC9165908; doi:10.1371/journal.pone.0269558)
Supplement: S2 Table — Chr = chromosome, ASR = alleles size range, FAM = 6-arboxyfluorescein dye, HEX = 6-Hexachloro-fluorescein dye. (DOCX) [file pone.0269558.s004.docx]

**S2 Table.** Microsatellite markers for linkage analysis of reported loci

| **Locus** | **Chr** | **Marker** | **Dye** | **ASR** | **Primer sequences (5’-3’)** | **Genbank** |
| --- | --- | --- | --- | --- | --- | --- |
| OTSC1 | 15 | D15S652 | FAM | 284-309 | F-GCAGCACTTGGCAAATACTC | G07893 |
|  |  |  |  |  | R-CATCACTCAAGGCTCAAGGT |  |
|  |  | D15S657 | HEX | 330-360 | F-TCTACATTGGACAGAAATGGG | G07903 |
|  |  |  |  |  | R- GATACACATTCTGATTCATGCG |  |
|  |  | D15S1004 | HEX | 247-271 | F-GGCAAGACTCCATCTCAAAA | Z53286 |
|  |  |  |  |  | R- GAATAAAAAGCCTGTAAACCACC |  |
| OTSC2 | 07 | D7S2513 | FAM | 157-181 | F-GCAGCATTATCCTCAACAGC | Z53958 |
|  |  |  |  |  | R-CACAAATGGCAGCCTTTC |  |
|  |  | D7S495 | FAM | 150-168 | F-TGGCATTCATTTACAATAGCC | Z16760 |
|  |  |  |  |  | R-AGCACCTGGTCCAATTTTCT |  |
|  |  | D7S2426 | HEX | 105-127 | F-GGTGGCTTGCCCAGAG | Z52266 |
|  |  |  |  |  | R-CCAGTGCATCTGTTTCCC |  |
| OTSC3 | 06 | D6S291 | FAM | 198-210 | F-CTCAGAGGATGCCATGTCTAAAATA | Z16904 |
|  |  |  |  |  | R-GGGATGACGAATTATTCACTAACT |  |
|  |  | D6S1660 | HEX | 203-217 | F-GAGTCTTGAGTAACTCCCACG | Z53853 |
|  |  |  |  |  | R-GACAATGAGTATCCCCCAC |  |
|  |  | D6S1680 | HEX | 176-198 | F-AAAATTCCACCCCCGC | Z51446 |
|  |  |  |  |  | R-CCATCTCCCCAGCAGAC |  |
| OTSC4 | 16 | D16S3025 | FAM | 90-110 | F-TCCATTGGACTTATAACCATG | Z52283 |
|  |  |  |  |  | R-AGCTGAGAGACATCTGGG |  |
|  |  | D16S515 | HEX | 222-244 | F-CATTCTGAAATTAGACAGCGATAGG | Z24558 |
|  |  |  |  |  | R-TGTGACCAGAGGCTTGC |  |
| OTSC5 | 03 | D3S1292 | FAM | 142-166 | F-TGGCTTCATCACCAGACC | Z16871 |
|  |  |  |  |  | R-CAGATTCAAGAGGCACTCCA |  |
|  |  | D3S3694 | HEX | 124-162 | F-AGTGTCCATCAACATGGG | Z54062 |
|  |  |  |  |  | R-TCGCACAAATAACAGGATTC |  |
|  |  | D3S1744 | FAM | 131-163 | F-TTTAAGCGGAAGGAAGTGTG | G08246 |
|  |  |  |  |  | R-CTGGCCCCATCTCTCTCTAT |  |
| OTSC7 | 06 | D6S300 | FAM | 188-207 | F-AGATACATTGACATTCTTCCGC | Z16991 |
|  |  |  |  |  | R-TCAAAAGCCAAAAGCCTACT |  |
|  |  | D6S1036 | FAM | 147-148 | F-CTTCATTCAAAGAGATAAATGGC | G09175 |
|  |  |  |  |  | R-GGATGGAATTATTCATGGCA |  |
|  |  | D6S406 | HEX | 186-206 | F-CCTGGGTGACAGAGTGAGAC | Z23304 |
|  |  |  |  |  | R-CACCATAGATTCTGAAGCACC |  |
| OTSC8 | 09 | D9S1844 | FAM | 215-257 | F-CATGCGAAAAACCGCTT | Z53591 |
|  |  |  |  |  | R-TCCGCAGCCTCAGAGA |  |
|  |  | D9S1777 | HEX | 235-247 | F-AGGCAATGCTGGTTCATAG | Z52124 |
|  |  |  |  |  | R-CTCCCAAATACCTGTTACCC |  |
|  |  | D9S1862 | HEX | 167-207 | F-CATGAGAGCACTGTATGAGGAC | Z53997 |
|  |  |  |  |  | R-ACATCAGGATTGTGGGTTC |  |
| RELN | 07 | D7S2509 | FAM | 270-290 | F-GTGGGAAACAGTATGATTGATT | Z51237 |
|  |  |  |  |  | R-CCTTTTTAAGGCTGAATAATGTGT |  |
|  |  | D7S2504 | FAM | 197-229 | F-TGTGGTACAATTTCAGACACATAA | Z53797 |
|  |  |  |  |  | R-CTGGAAACCAGTGTTTTCACTT |  |
|  |  | D7S796 | FAM | 162-198 | F-TTTTGGTATTGGCCATCCTA | G08623 |
|  |  |  |  |  | R-GAAAGGAACAGAGAGACAGGG |  |

# Chr = chromosome, ASR = alleles size range, FAM = 6-arboxyfluorescein dye, HEX = 6-Hexachloro-fluorescein dye
