# Supplementary material for: The risks of RELN polymorphisms and its expression in the development of otosclerosis
Source: PLoS One. 2022 Jun 3;17(6):e0269558. doi: 10.1371/journal.pone.0269558 (PMC9165908; doi:10.1371/journal.pone.0269558)
Supplement: S3 Table — F, forward primer; R, reverse primer; bp, base pair. (DOCX) [file pone.0269558.s005.docx]

**S3 Table.** Semi quantitative PCR and real-time PCR primers used for gene expression analysis

| **Genes** | **Primer sequence** | **Annealing temperature (°C)** | **Product Size (bp)** |
| --- | --- | --- | --- |
| *RELN* | F: 5’CAGGCATCACAGAGCATTGGAG 3’ | 61 | 130 |
|  | R:5’ TGAGGTTGGTTGTGGGCAGGT 3’ |  |  |
| *VLDLR* | F: 5’ TCTGTTGGACACACGTACCC 3’ | 58 | 88 |
|  | R:5’ CCTCAAAGGTCAACATTTGTCA 3’ |  |  |
| *LRP8* | F: 5’ CTGATGGCTCCGATGAGTC 3’ | 58 | 73 |
|  | R: 5’ GGTCCACAGCTCAGCTTCTC 3’ |  |  |
| *18S rRNA* | F: 5’ GTAACCCGTTGAACCCCATT 3’ | 56 | 151 |
|  | R: 5’ CCATCCAATCGGTAGTAGCG 3’ |  |  |

F, forward primer; R, reverse primer; bp, base pair.
