# Supplementary material for: The risks of RELN polymorphisms and its expression in the development of otosclerosis
Source: PLoS One. 2022 Jun 3;17(6):e0269558. doi: 10.1371/journal.pone.0269558 (PMC9165908; doi:10.1371/journal.pone.0269558)
Supplement: S4 Table — Notes: *comprised European population from the genome wide association study (DG: Discovery group, RS1: Replication set 1, RS2: Replication Set 2). **comprised four cohorts: German, Italian, Swiss and Romanian. (DOCX) [file pone.0269558.s006.docx]

**S4 Table.** Characteristics of the eligible studies used for meta-analysis

| **First Author** | **Ethnic group** | **Case** | **Control** | **OR** | ***P* value** |
| --- | --- | --- | --- | --- | --- |
| Schrauwen I et al, (2009)* | European (DG) | 302 | 302 | 1.82 | 0.00001 |
| Schrauwen I et al, (2009)* | European (RS1) | 392 | 392 | 1.480 | 0.003 |
| Schrauwen I et al, (2009)* | European (RS2) | 455 | 480 | 1.430 | 0.003 |
| Schrauwen I et al, (2010)** | German | 265 | 206 | 1.245 | 0.269 |
| Schrauwen I et al, (2010)** | Italian | 119 | 150 | 1.338 | 0.189 |
| Schrauwen I et al, (2010)** | Swiss | 102 | 101 | 1.321 | 0.259 |
| Schrauwen I et al, (2010)** | Romanian | 105 | 93 | 1.296 | 0.331 |
| Khalfallah A et al, (2010) | Tunisian | 149 | 152 | 1.392 | 0.060 |
| Sommen M et al, (2014) | Hungary | 153 | 300 | 0.904 | 0.545 |
| Mowat A J et al, (2018) | British | 374 | 374 | 1.160 | 0.330 |
| This Study | Indian | 254 | 262 | 0.569 | 0.004 |

Notes: *comprised European population from the genome wide association study (DG: Discovery group, RS1: Replication set 1, RS2: Replication Set 2). **comprised four cohorts: German, Italian, Swiss and Romanian.
