# Supplementary material for: The risks of RELN polymorphisms and its expression in the development of otosclerosis
Source: PLoS One. 2022 Jun 3;17(6):e0269558. doi: 10.1371/journal.pone.0269558 (PMC9165908; doi:10.1371/journal.pone.0269558)
Supplement: S5 Table — (DOCX) [file pone.0269558.s007.docx]

**S5 Table.** Two-point LOD score estimation for known OTSC loci in a family

| **Locus** | **Marker** | **Two point LOD score at θ =** | | | | |
| --- | --- | --- | --- | --- | --- | --- |
|  |  | **T0.000** | **T0.100** | **T0.200** | **T0.300** | **T0.400** |
| OTSC1 | D15S652 | 0.9018 | 0.7195 | 0.5213 | 0.3158 | 0.1282 |
|  | D15S1004 | -100000 | -0.8642 | -0.5330 | -0.3830 | -0.2040 |
|  | D15S657 | -100000 | -1.1221 | -0.4684 | -0.2036 | -0.0833 |
| OTSC2 | D7S495 | -100000 | -0.1273 | 0.2099 | 0.2667 | 0.1836 |
|  | D7S2513 | -100000 | -1.3385 | -0.5993 | -0.2513 | -0.0752 |
|  | D7S2426 | -100000 | -0.2347 | -0.0808 | -0.0521 | -0.0479 |
| OTSC3 | D6S1660 | -100000 | -0.3738 | 0.0216 | 0.1411 | 0.1230 |
|  | D6S1680 | -100000 | -2.0270 | -0.9784 | -0.4487 | 0.1500 |
| OTSC4 | D16S3025 | -100000 | -0.6321 | -0.1835 | -0.0053 | 0.0437 |
|  | D16S515 | -100000 | -0.6784 | -0.2746 | 0.1279 | 0.0656 |
| OTSC5 | D3S1292 | -100000 | 0.1185 | 0.2257 | 0.2873 | 0.2022 |
|  | D3S3694 | -100000 | 0.1185 | 0.2257 | 0.2873 | 0.2011 |
|  | D3S1744 | -100000 | 0.1884 | 0.0103 | 0.0704 | 0.0615 |
| OTSC7 | D6S1036 | -100000 | -2.5375 | -1.3867 | -0.7409 | -0.3084 |
|  | D6S406 | -100000 | -2.3580 | -1.2381 | -0.6310 | -0.2469 |
|  | D6S300 | -100000 | -0.2347 | -0.0808 | -0.0521 | -0.0479 |
| OTSC8 | D9S1844 | -100000 | -3.9355 | -2.1825 | -1.1846 | -0.5022 |
|  | D9S1862 | 0.00000 | 0.0000 | 0.0000 | 0.0000 | 0.0000 |
|  | D9S1777 | -100000 | -2.0969 | -1.1938 | -0.6655 | 0.2907 |
