# Supplementary material for: The risks of RELN polymorphisms and its expression in the development of otosclerosis
Source: PLoS One. 2022 Jun 3;17(6):e0269558. doi: 10.1371/journal.pone.0269558 (PMC9165908; doi:10.1371/journal.pone.0269558)
Supplement: S1 Raw images — (PDF) [file pone.0269558.s008.pdf]

## Original blot and gel images

### Fig 4A. Full-length gels images contained in the manuscript’s main figures

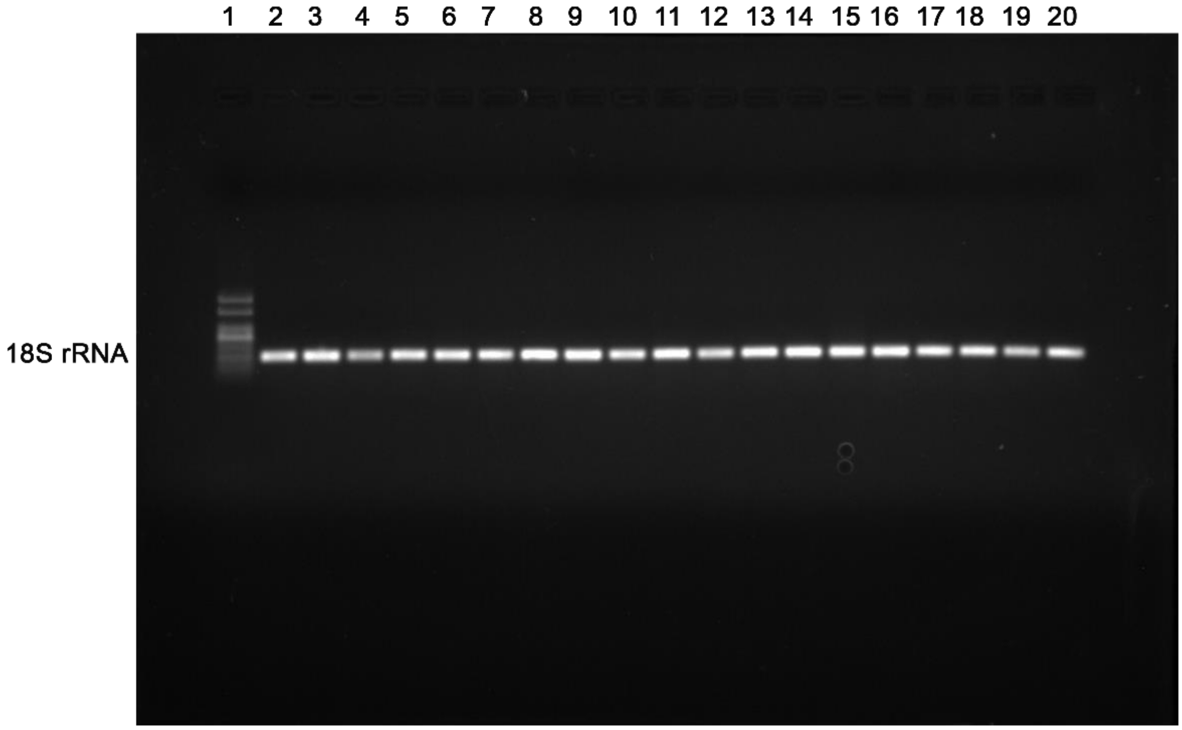


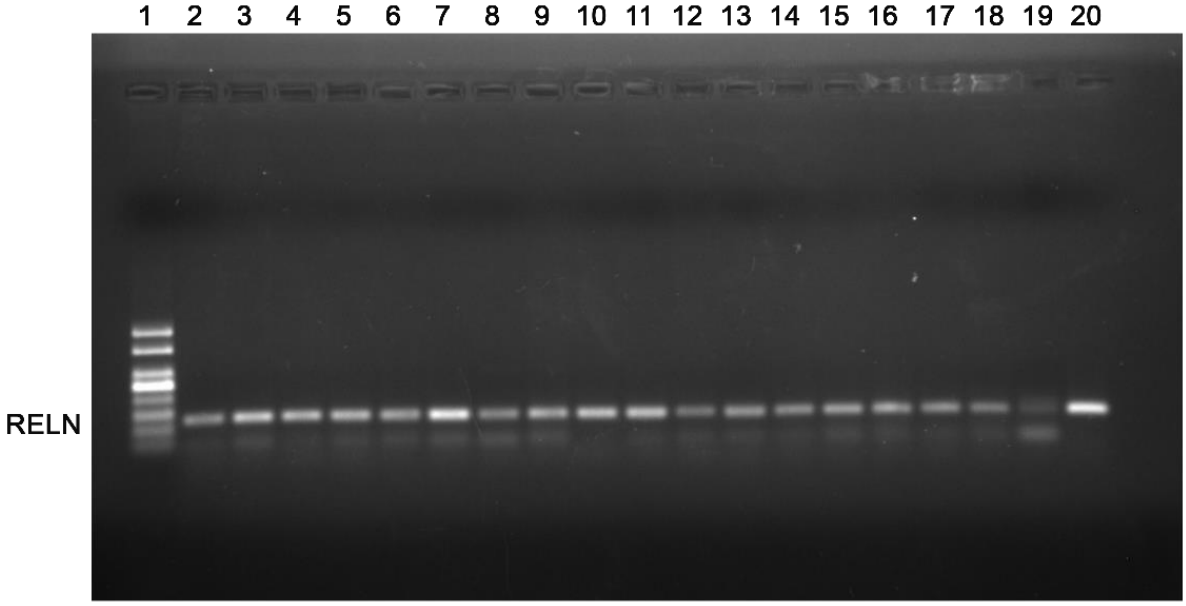

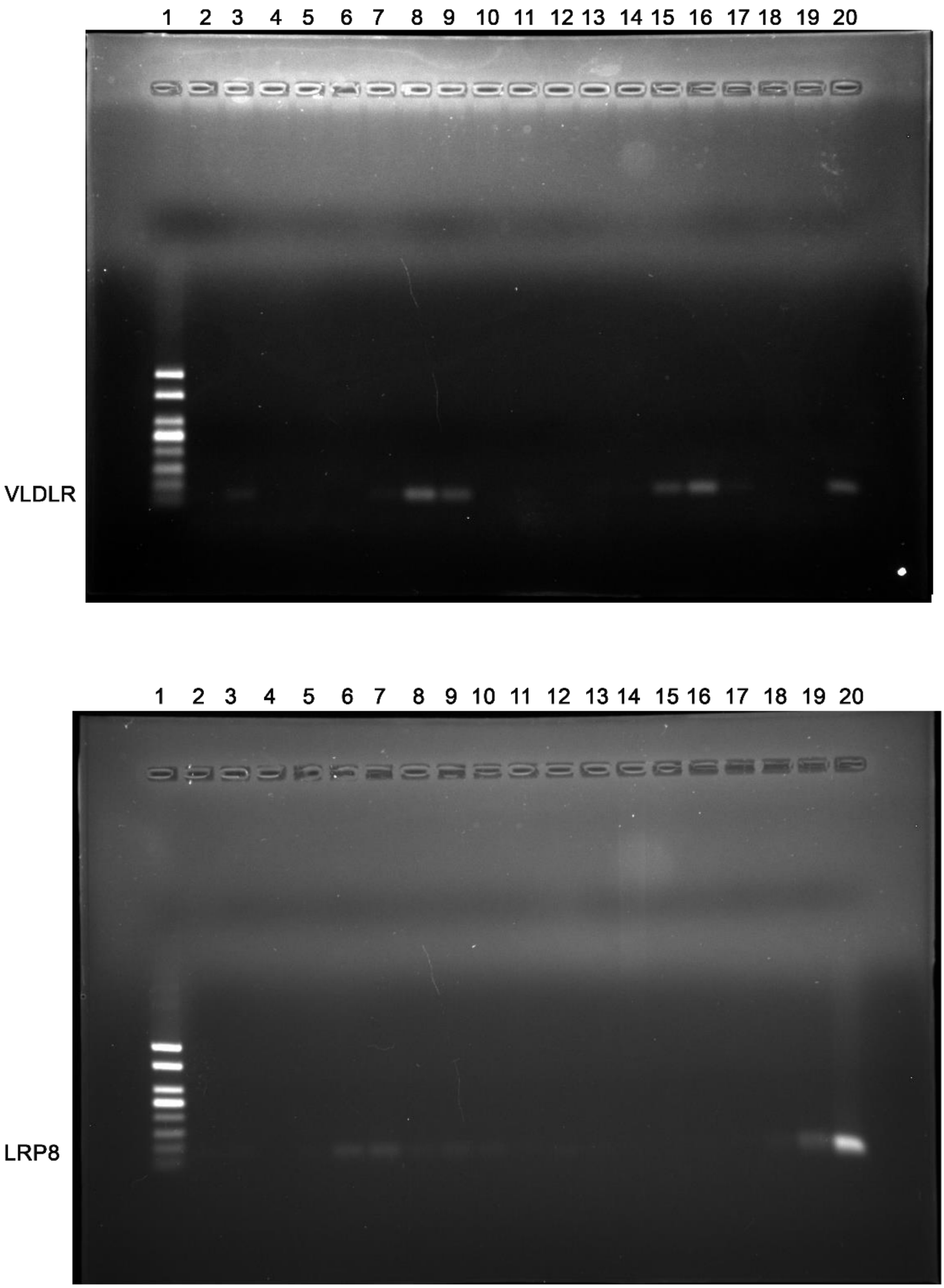


**Fig 4:** Uncropped gels corresponding to Fig 4A.

# Fig 5A and B. Full-length blot images contained in the manuscript's main figures

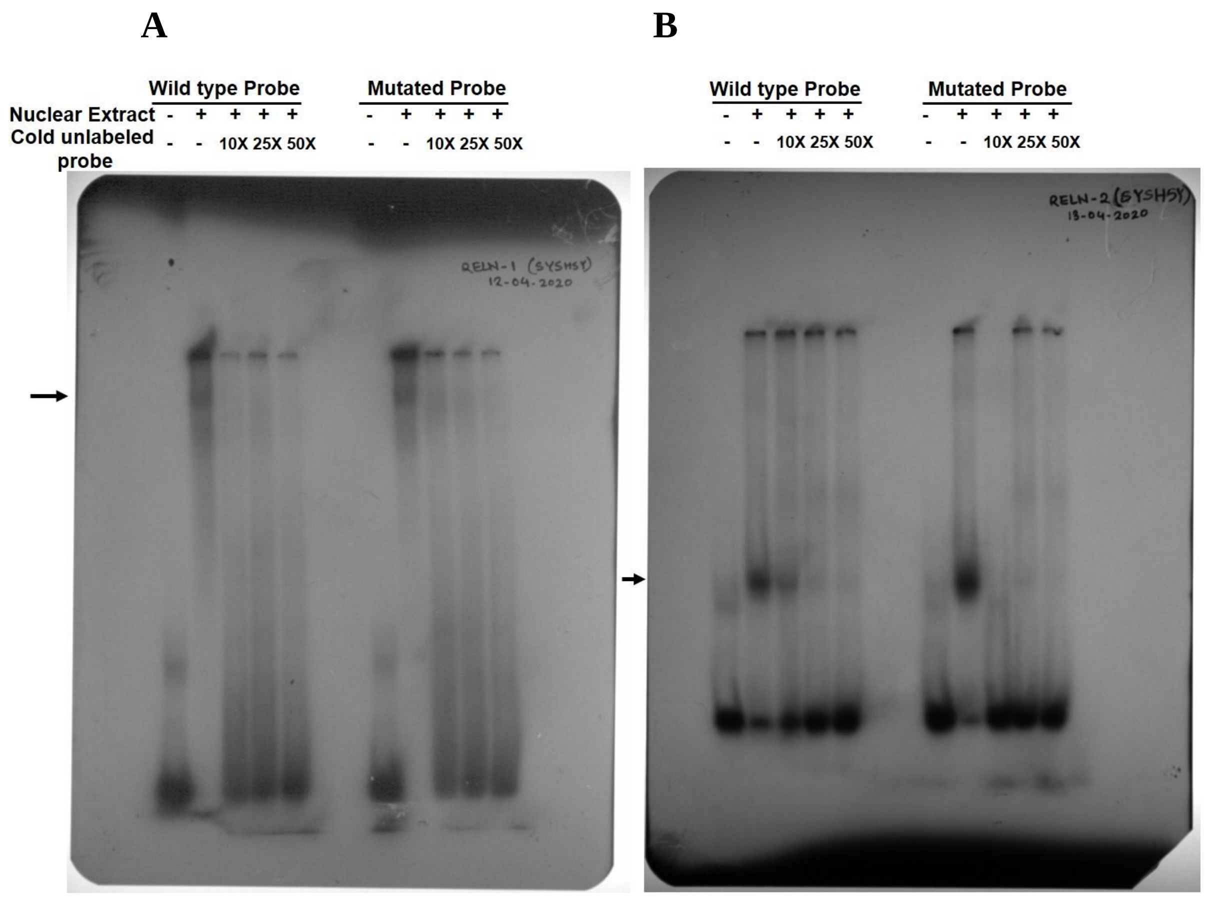


**Fig 5:** Uncropped blot corresponding to Fig 5A and 5B.

# Fig 6A-D. Original uncropped images contained in the manuscript's main figures

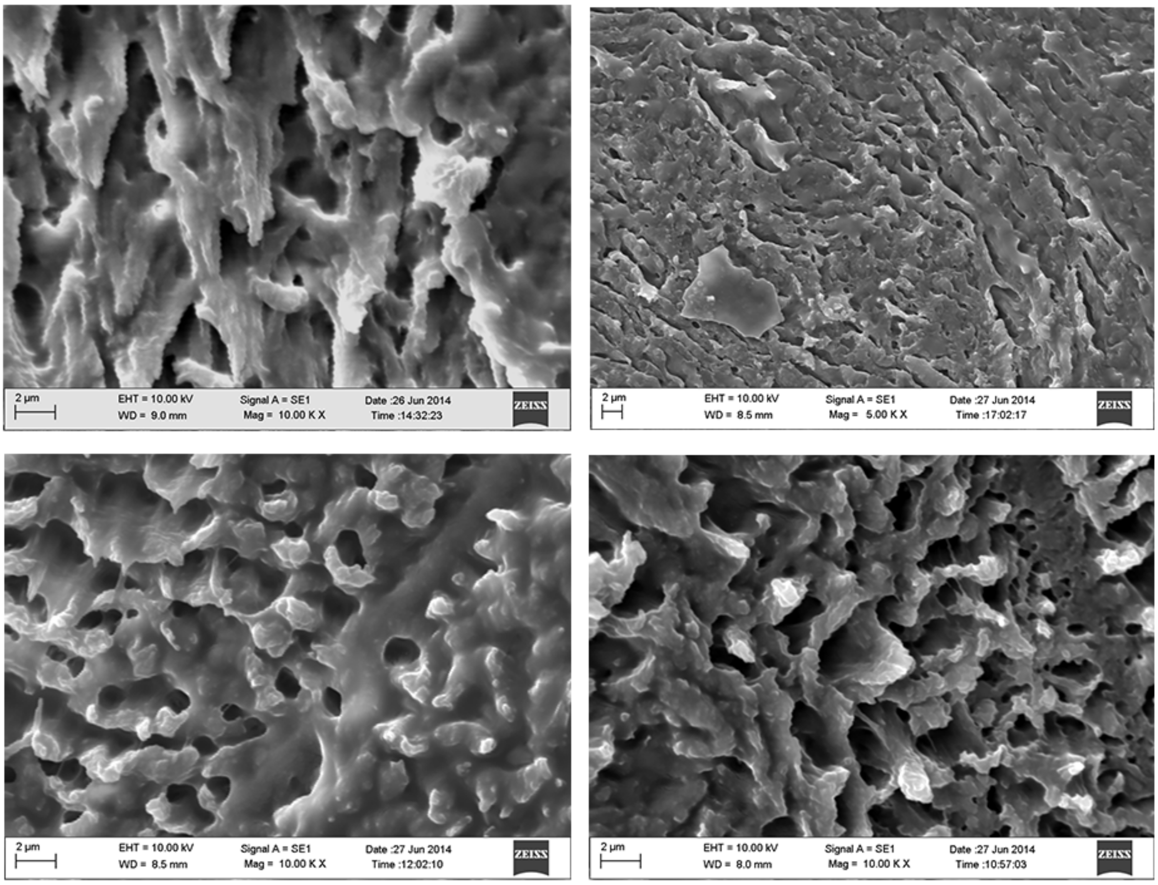


**Fig 6:** Original image corresponding to Fig 6A,6B, 6C and 6D.

# Fig 7A to D. Full-length original image with visible edges

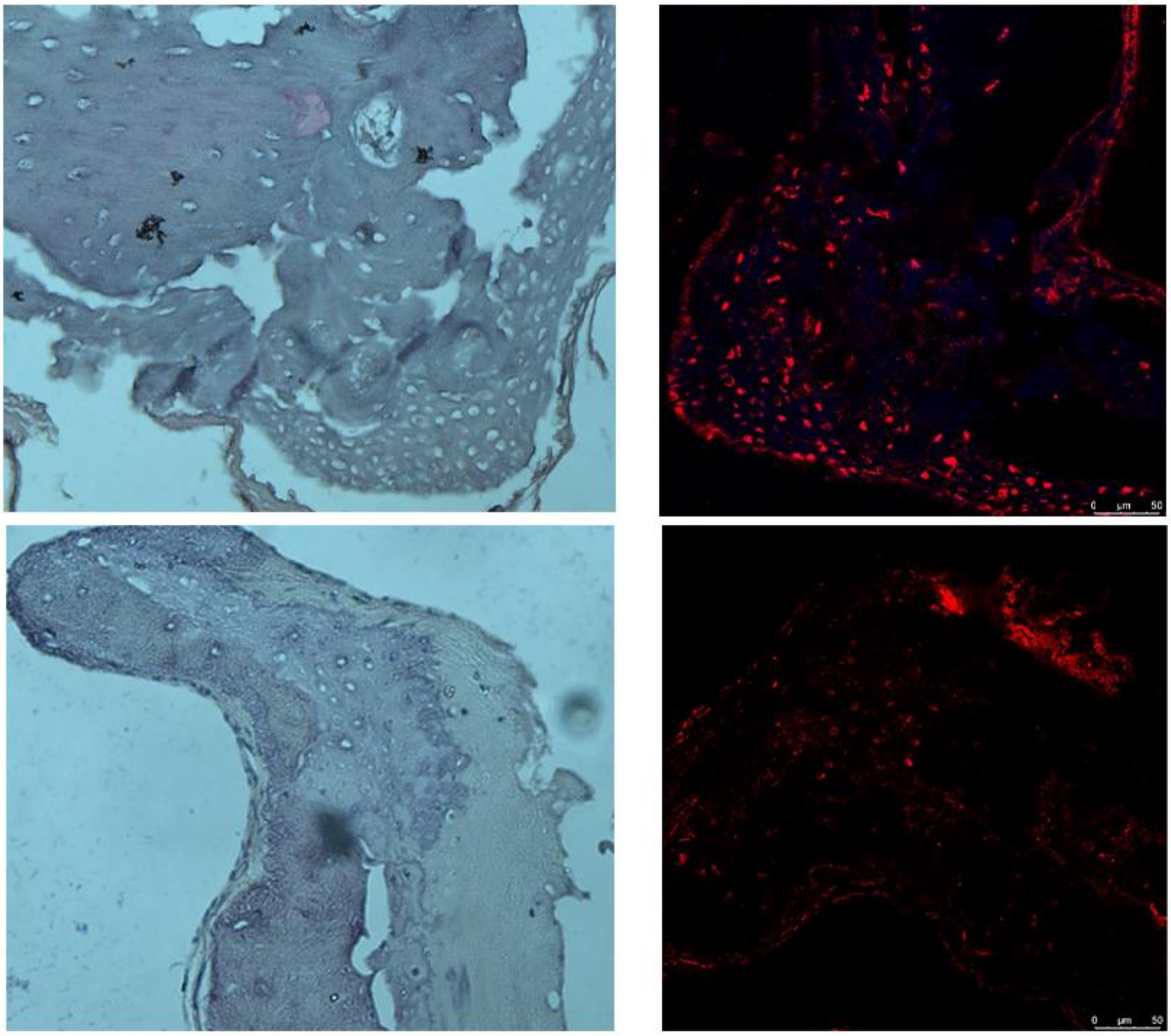


**Fig 7:** Original image corresponding to Fig 7A to 7D

## S1 Figs. Full-length images contained in the supplemental figures.

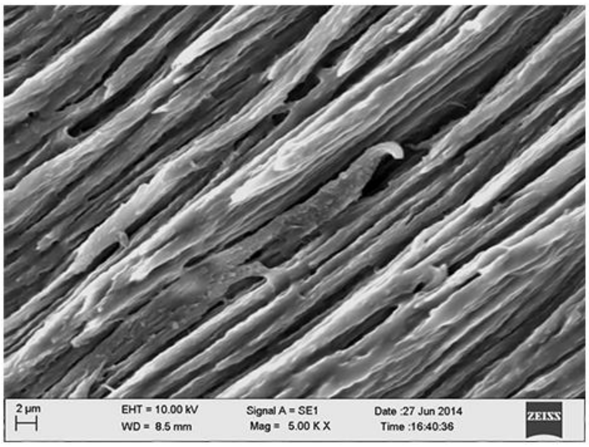


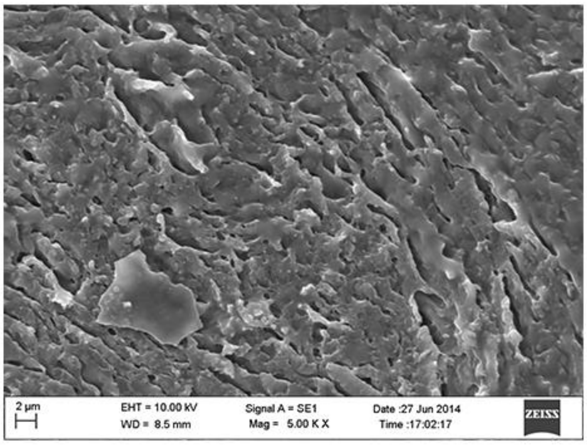


**S1 Figs:** Original image corresponding to S1 Fig.

**S2 Figs. Full-length images contained in the supplemental figures.**

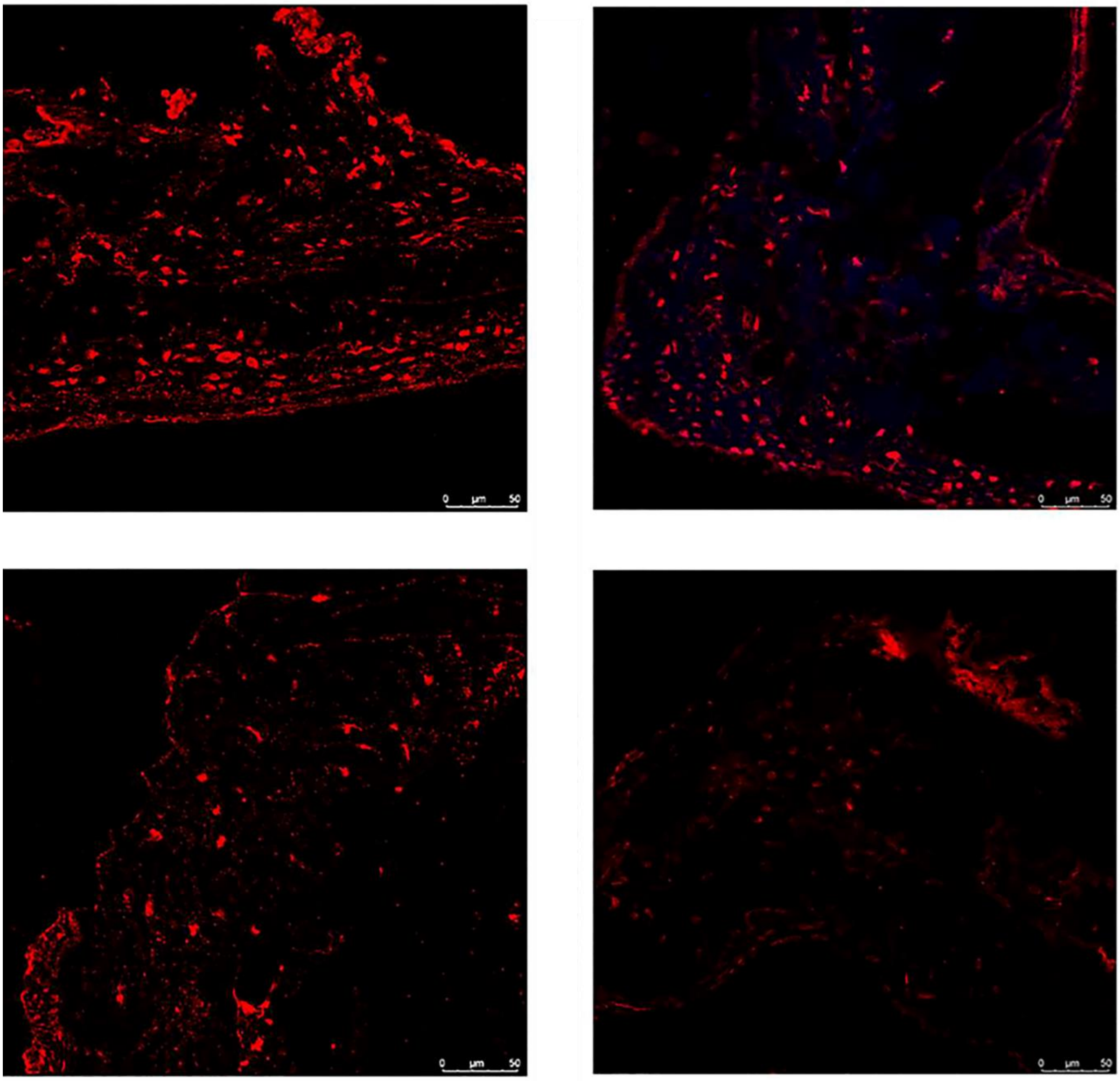


**S2 Figs:** Original image corresponding to S2 Fig.
